# Finite Element Simulation of Interstitial–Lymphatic Fluid Flow and Nanodrug Transport in a Solid Tumor: An Intratumoral Injection Approach

**DOI:** 10.34133/bmef.0119

**Published:** 2025-06-06

**Authors:** Gobinda Debnath, Buddakkagari Vasu, Rama Subba Reddy Gorla

**Affiliations:** ^1^ Department of Mathematics, Motilal Nehru National Institute of Technology Allahabad, Prayagraj 211004, UP, India.; ^2^ Department of Aeronautics and Astronautics, Air Force Institute of Technology, Wright Patterson Air Force Base, Dayton, OH 45433, USA.

## Abstract

**Objective:** This study presents a mathematical model and finite element simulations to investigate interstitial fluid flow and nanodrug transport in a solid tumor, incorporating transvascular exchange, convection–diffusion–reaction dynamics, and intratumoral injection mechanisms. **Impact Statement:** Optimizing nanodrug distribution remains a critical challenge in cancer therapy. The proposed model advances nanomedicine by enhancing the mechanistic understanding of nanodrug transport in a solid tumor. **Introduction:** Cancer, a global threat, often manifests as solid tumors driven by uncontrolled cell growth. The heterogeneous microenvironment, lymphatic drainage, nano-bio interactions, and elevated interstitial fluid pressure (IFP) hinder effective nanodrug delivery. Nanoparticle (NP)-based drug delivery systems offer a promising solution, with FES providing an effective approach to model and simulate the complex delivery process. **Methods:** The model considered a spherical and symmetrical tumor architecture comprising a central necrosis region, viable tumor, and surrounding healthy tissue with functional lymphatic dynamics. Substantial nanodrug carriers (dextran, liposomal, polyethylene glycol (PEG)-coated gold, and magnetic) and conventional doxorubicin are evaluated in the tumor. The governing fluid flow and solute transport equation along with the specified boundary conditions are solved using the finite element method through the Galerkin approach. **Results:** Simulations show that IFP peaks in the necrotic core and sharply declines at the viable–healthy tissue interface. Both fluid pressure and velocity are sensitive when fluid flow resistance drops below 5. Necrotic core size influences IFP, and critical necrotic radius (*R*_CN_) marks pressure stabilization and defines the threshold for effective nanodrug delivery. Vascular normalization and functional lymphatic dynamics show marginal impact. Smaller NPs (~10 nm) diffuse faster but undergo rapid degradation, while larger particles (>30 nm) exhibit prolonged retention at the injection site. Liposomal, PEG-coated gold, and magnetic variants demonstrate superior therapeutic action compared to conventional doxorubicin. **Conclusion:** The findings of the study highlight its strong potential for optimizing nanodrug delivery and design, as well as hyperthermia treatment, enhancing personalized cancer therapy.

## Introduction

Cancer, a formidable threat in modern life, extends its reach globally, affecting millions of lives. The majority of human cancers manifest as solid tumors driven by uncontrolled cell proliferation [1]. Estimates suggest that in the year 2024, approximately 2,001,140 new cases of cancer will be diagnosed in the United States alone [2]. Additionally, it is projected that around 611,720 individuals will lose their lives due to cancer-related causes, amounting to an average of 50,000 deaths per month. The primary methods for treating cancer include surgical removal, chemotherapy, and radiation therapy. However, surgery is only feasible for tumors that are easily accessible, chemotherapy comes with substantial side effects, and radiation therapy can harm healthy tissues [3]. Anticancer drugs can be administered orally, intravenously, or directly into the tumor (intratumoral). The effectiveness of these treatments depends on the drug’s ability to reach the tumor, convert into its active form, and be absorbed at the target site [4]. Unfortunately, many drugs lose their potency due to metabolism, degradation before reaching the tumor, excretion, and unintended binding to nontarget areas [4].

A major challenge in cancer treatment is that drugs often struggle to reach tumors effectively due to the chaotic nature of tumor blood vessels [5]. Abnormal vascular structures, highly permeable vessels, and lack of functional lymphatics elevate interstitial fluid pressure (IFP), hindering fluid and nanodrug distribution and complicating therapeutic delivery [6]. In vivo experiments to comprehend drug delivery complexities are complex, leading to the development of mathematical models as important tools for investigation. The foundational work of Baxter and Jain [7–9], who introduced the mathematical model calculating IFP, interstitial fluid velocity (IFV), and drug concentration in solid tumors, marked a pivotal moment in understanding the complex interplay of factors influencing drug delivery. Building upon these foundational 1D models, Tan et al. [10] advanced the field by developing a simulation framework specifically dedicated to drug delivery for brain tumor. Their focus centered on unraveling the dynamics of drug convection and diffusion, providing nuanced insights into the kinetics governing these processes. Further, the scientific understanding has been expanded by introducing a fluid flow mathematical model, utilizing it to scrutinize critical aspects such as tumor size, shape, and the formation of necrotic zones [11]. Pishko et al. [12] notably contributed to this discourse by introducing a model that encompasses tumors with spatially varying tissue transport properties, adding a layer of complexity to the understanding of these dynamics. Kashkooli et al. [13] conducted a comprehensive exploration into the impact of vascular normalization (VN) on fluid dynamics and drug diffusion, particularly in the context of therapeutic drugs administered through intravenous injection. Their investigation unveiled the substantial influence of VN on both drug and pressure distribution, shedding light on critical dimensions of drug delivery. Research has shown that several factors impact how drugs are released into the extracellular space of tumors and efficiency of drug delivery. These factors include the microvascular structure [5], fluid flow resistance [14], reflection coefficient, diffusivity, permeability, pore size, drug clearance rate, and the overall drug characteristics [15,16].

Nanodrug delivery in tumors utilizes nanoparticles (NPs) as carrier to transport therapeutic agents directly to tumor sites, leveraging technological advancements [17]. An optimal NP design for effective drug delivery in the tumor microenvironment (TME) must consider several critical parameters, including size, shape, surface properties, and drug release profile [16]. The size of NPs plays a vital role in their transport and uptake [18]. Smaller NPs exhibit higher diffusion rates, facilitating deeper tumor penetration, while larger NPs may face steric hindrances and reduced mobility through the tumor’s extracellular matrix [19]. Similarly, the shape of NPs substantially influences their interaction with biological systems. Spherical NPs are better suited for marginalization and deeper penetration [20], whereas rod-shaped NPs exhibit superior adhesion to the vascular walls [21], enhancing their retention and localized delivery efficiency. Surface functionalization, such as charge modulation and hydrophilicity, is also crucial to mitigate immune system recognition, prolong systemic circulation, and improve tumor targeting [22]. The enhanced permeability and retention (EPR) effect [23], though a cornerstone of NP-based cancer therapy, is often hindered by the tumor’s heterogeneous vasculature and elevated IFP, which impede uniform NP distribution. Moreover, the local drug concentration within a tumor plays a critical role in designing NPs for effective drug delivery [24]. Intratumoral drug distribution involves multiple interconnected processes, including diffusion, convection, and reaction, which govern how NPs traverse the TME and interact with cells [15]. The molar concentration of the drug markedly impacts the binding and internalization of NPs within the tumor spheroid, highlighting the importance of precise parameter tuning [25]. Mathematical models have potential to simulate nanodrug distribution in tumors, considering structural changes and necrotic regions [26]. Computational tools bridge theoretical design and experimental validation, optimizing NPs for efficacy and reduced off-target effects [16]. These simulations enhance nanodrug delivery, particularly to necrotic cores, where conventional methods fall short. Chen et al. [16] reviewed advancements in nanomedicine, emphasizing NP properties, tumor environment, and targeting strategies to improve tumor accumulation and clinical applicability.

Taheri-Ledari et al. [27] developed an efficient strategy for cancer therapy involving the pretreatment of tumor masses with calcium hydroxide, followed by the design of an advanced delivery system utilizing iron oxide NPs for the delivery of Taxotere (TXT). The study also conducted in vitro, ex vivo, and in vivo experiments to validate the efficacy of the treatment. Goodman et al. [28] examined the NP diffusion within 3-dimensional (3D) tumor spheroids, probing the efficacy of NPs as carrier for therapeutic drugs. Intriguingly, mouse models were subjected to intravenous ferrofluid injections to scrutinize NP tolerance [29]. A parallel study by Singh et al. [30] explored the impact of mild hyperthermia on the transport properties of tumors, coupled with the distribution dynamics of intravenously administered gold NPs. Sefidgar et al. [31] examined the temperature distribution in necrotic tumors and surrounding tissue during magnetic hyperthermia. The study evaluated the efficiency of intravenous bolus injection for systemic drug delivery by examining the effects of necrotic regions on drug and temperature distribution. Only 15.5% of the MNP concentration was found to reach the necrotic area in tumors where necrosis occupied 50% of the radius, which led to a decrease in heat generation. Wilhelm et al. [32] reported that roughly 1 out of every 100 NPs administered intravenously accumulates in the tumor. Furthermore, Dai et al. [33] observed that, out of these tumor-accumulated NPs, only 1 in 50 manages to enter cancer cells. Chou et al. [34] examined a one-dimensional (1D) mathematical model to assess the effectiveness of cancer treatment by studying the distribution of NP dosages in tumors. The study found that drug transport properties were affected by both vascular density and NP size. Suleman and Riaz [35,36] explored the use of core-shell magnetic NPs (MNPs) for liver and breast cancer treatment through hyperthermia. It compared temperature generation from concentration-dependent heat sources with that from a constant heat source, aiming to determine the most efficient heating mechanism via finite element method (FEM) modeling simulation. Tumors amenable to intratumoral injection also present an opportunity for surgical removal [15]. However, precise delineation of tumor boundaries and the risk of incomplete tumor extraction or inadvertent damage to healthy tissue constitute formidable challenges in surgical interventions. Recognizing these concerns, various studies [15,19,37,38] have elucidated the distinct advantages associated with intratumoral injection, positioning it as a strategic alternative to conventional administration methods. Intratumoral administration has demonstrated its efficacy as a valuable approach for delivering therapeutic doses of diverse NPs like nanodrugs and nanocarriers directly into tumors through the injection of nanofluid [38]. Mahesh et al. [15] developed a finite element simulation (FES) model to predict the interstitial fluid flow dynamics and NP distribution through an intratumoral injection approach, taking into account the influence of transvascular exchange of interstitial fluid. The study observed that smaller NPs exhibit faster diffusion and rapid degradation, while larger particles are retained for longer periods. Moreover, Mahesh et al. [38] extended the previous model [15] and incorporated heat transfer equation for simulating the clinical procedure of MNP hyperthermia. It aids in evaluating the accuracy of predicting tumor temperatures and treatment efficiency in the ideal case of confined uniform distribution of nanodrugs. Hosseinpour et al. [19] explored the potential of combining low-intensity ultrasound with intratumoral injection of drug-loaded MNPs for chemotherapy and thermal necrosis using a sophisticated FES model. The study found that smaller MNPs demonstrated enhanced diffusion and deeper tumor penetration but were rapidly cleared into the bloodstream, while larger MNPs produced greater heat due to their increased responsiveness to sound waves.

### Lymphatics and fluid flow resistance

Lymphatic capillaries, absent in necrotic (dead tissue part of the tumor) and viable (proliferating part of a tumor with functional blood vessels) regions [6], play a key role in fluid homeostasis by absorbing interstitial fluid and preventing swelling [39]. Unlike circular blood vessels, lymphatics have an elliptical cross-section due to low hydrostatic pressure (−4 to −6 mmHg) and operate similarly to veins (act as sucking pump), using a pumping mechanism [39]. In contrast, interstitial hydrostatic pressure varies from −8 to 6 mmHg, with tumor tissues typically showing higher pressures [40,41]. The negative interstitial pressures observed in subcutaneous and other tissues are predominantly attributed to the orchestrated action of lymphatics [40,41].

Fluid flow resistance, driven by interstitial and vascular resistance, markedly impacts therapeutic delivery in tumors. Interstitial resistance arises from abnormal vasculature, elevated IFP, dense extracellular matrix, cellular components, and the absence of functional lymphatics [4]. Vascular resistance, influenced by tumor vasculature abnormalities, hampers oxygen, nutrient, and drug delivery [42,43]. Anti-angiogenic agents offer a promising strategy to normalize vasculature, reduce IFP, and enhance perfusion [1].

### Review of FES models on interstitial fluid flow and nanodrug transport

FES has become an essential computational tool for studying complex biological systems, particularly in modeling interstitial fluid flow and nanodrug transport within solid tumors [15,19]. By discretizing the domain into smaller elements and employing numerical methods, FES enables the simulation of complex physical phenomena [44]. Unlike analytical approaches that often rely on simplified geometries and assumptions, FES offers the flexibility to incorporate realistic tumor structures, heterogeneous material properties, and coupled nano-bio interactions such as nanodrug phagocytosis due to the presence of tumor-associated macrophages (TAMs) [15,45].

Over the years, numerous FES models have been developed to investigate interstitial fluid flow and nanodrug transport in solid tumors. These models vary widely in their objectives, assumptions, mathematical formulations, and numerical implementations. In order to better understand the current state of the field and identify unresolved challenges, a systematic literature review is conducted, focusing on studies with strong relevance to the current research. This review analyzes studies published between 2011 and 2024, sourced from databases such as Google Scholar, Web of Science, ScienceDirect, PubMed, etc. Using search terms like “Interstitial Fluid Flow”, “Nanodrug Transport”, “Tumor”, and “Finite Element Simulation”, several benchmark original studies are identified and thoroughly reviewed. Table 1 summarizes the key methodologies, findings, and limitations of the studies, highlighting variations in tumor geometry modeling, governing equations, boundary conditions, and software implementations (with elements count). This structural comparative review not only contextualizes the current state of research but also paves the way for the contributions of this study, which aims to advance tumor drug delivery modeling through innovative FES approaches.

**Table 1.** Comparison of FES models for interstitial fluid flow and nanodrug transport in solid tumors

Study	Objective and key assumptions	FES methodology			Findings	Key limitations
		Geometry	Governing equations and BCs	Software		
Su et al. [48], (2011)	Fluid transport; a poroelastic model, tissue deformation due to fluid flow	2D axis-symmetric circular disc shape of tumor domain	Darcy’s law (DL) and Navier–Stokes equation (NS); Dirichlet BC at outer edge: pressure and velocity set to zero	COMSOL Multiphysics (FEM); 22,792 elements	Predicted nanofluid fluid flow and induced tissue deformation.	Neglected diffusion transport
Tang et al. [49], (2018)	Solute transport; interstitium as an isotropic porous media, intratumoral injection	3D spherical tumor surrounded by normal tissue	DL and convection-diffusion equation (CD); initial concentration of NPs in all tissues is zero and no flux outer BC	COMSOL Multiphysics (FEM)	Predicted NP distribution for different diffusion durations	Overlooked necrotic core, transvascular exchange, and reaction term
Suleman and Riaz [35], (2020)	Interstitial fluid flow and solute transport; intratumoral injection of NP	2D artificial liver geometry with a 10-mm tumor diameter	DL and CD; zero pressure and concentration at the outer boundary	COMSOL Multiphysics (FEM); 70,815 elements	Estimated IFP, IFV, and solute distribution	Ignored nano-bio interactions and NPs transvascular exchange
Suleman and Riaz [36], (2020)	Interstitial fluid flow and solute transport; direct needle injection of magnetic nanofluid	A 3D model of a breast tumor (10 mm radius), surrounded by normal tissue (30 mm radius)	DL and CD; ICs set velocity and pressure to zero in normal and tumor breast tissues, with no-flow boundaries and zero concentration at the outer normal tissue.	COMSOL Multiphysics (FEM); 350,668 elements	Predicted IFP and IFV along the axes and positional solute distribution	High computational cost; ignored NPs reaction rate term
Suleman and Riaz [75], (2020)	Interstitial fluid flow and solute transport; realistic blood vessels dynamics, EPR effect	2D realistic geometry featuring blood vessel, epithelial cells, and cancer cells in arrays.	NS and CD; zero IC for velocity, and concentration, Dirichlet inlet velocity and zero-flux BC for concentration	COMSOL Multiphysics (FEM); 116,832 elements	Estimated nanofluid velocity and concentration	Ignored nanofluid pressure distribution and reaction rate
Sarraf et al. [69], (2021)	MNPs transport trajectory in tumor; heterogeneous nature of healthy and tumors capillary vascular network	2D tumor domain with capillary vascular network	NS; a uniform inlet velocity and constant outlet pressure are applied, with no-slip conditions at walls and fluid-particle interfaces	COMSOL Multiphysics (FEM)	Evaluates MNPs targeting performance via magnetic field distribution capture	Did not consider NP concentration distribution
Mahesh et al. [15], (2022)	Interstitial fluid flow and solute transport; presence of transvascular and translymphatic exchange of NPs, degradation of NPs	3D spherical tumor with necrotic, viable, and healthy tissue region	DL and convection–diffusion–reaction (CDR) equation; pressure set to zero at outer boundary, initial concentration is set to zero across the domain, with an open boundary condition at the outer boundary	COMSOL Multiphysics (FEM); 363,692 elements	Showed spatial and temporal distribution of IFV, IFV and solute	High computation cost; incomplete lymphatic dynamics
Caddy et al. [76], (2022)	Nanoparticle transport; Deposition of NPs onto tumor cells	3D idealized spherical tumor	NS and CDR; velocity set zero as to eliminate convection and at outer edge: pressure set to zero, zero-flux concentration	COMSOL Multiphysics (FEM)	Predicted concentration on fluid and deposition of charged particles on cell surface	Neglected convection, porosity, and degradation of NPs
Mahesh et al. [38], (2023)	Fluid and solute transport; nanoparticle infusion, diffusion, intratumoral injection	3D spherical tumor with necrotic, viable, and healthy tissue region	DL and CD; A Dirichlet BC is applied for pressure, initial concentration is set to zero across the domain, with an open boundary condition at the outer boundary	COMSOL Multiphysics (FEM); 666,578 elements	Showed spatial and temporal distribution of IFV, IFV and solute	Incomplete lymphatic dynamics; ignored NPs reaction in TME
Mohammadi et al. [47], (2023)	Interstitial fluid flow and solute transport; realistic vascular network with intravenous injection	Synthetic 2D tumor geometry with a modeled microvascular network	DL and CDR; IFP at the outer boundary is set to 0 Pa, with an open boundary for concentration. At the tumor–normal interface, variables are continuous	COMSOL Multiphysics (FEM)	Displayed the IFP, IFV, and anti-angiogenic agent distribution	Neglected solute degradation due to nano-bio interaction
Mahesh et al. [67], (2023)	Interstitial fluid flow and solute transport; uniform and Gaussian distribution of MNPs	3D realistic breast tumor geometry composed of necrotic, viable, and healthy tissue	DL and CD; pressure is set to zero at outer boundary, initial concentration is set to zero across the domain, with an open boundary condition at the outer boundary	COMSOL Multiphysics (FEM); 580,684 elements	Predicted MNP drug carrier for given magnetic and NP parameters	High computational cost and ignored solute degradation due to nano-bio interaction
Rezaeian et al. [77], (2023)	Interstitial fluid flow and solute transport; vascularized tumor with intraperitoneal (IP) chemotherapy	2D tumor geometry derived from a vascularized tumor image.	DL and CDR; concentration at the tumor surface is fixed at 0.8 mol/m^3^. Inlet and outlet pressures are set at 25 and 10 mmHg, respectively, with zero pressure at outer boundary	COMSOL Multiphysics (FEM); 125,034 elements	Predicted intravascular blood pressure and velocity and nanodrug concentration	Drug concentration was constant at outer edge of tumor
Hosseinpour et al. [19], (2024)	Fluid flow and drug (free and bound) transport; tumor with non-uniform microvasculature structure	3D spherical tumor separating into 3 regions: proliferation, quiescent, and hypoxia	DL and CDR; pressure is set to zero at outer boundary, initial concentration is set to zero across the domain, with an open boundary condition at the outer boundary	COMSOL Multiphysics (FEM)	Displayed the spatial and temporal distribution of drug-loaded magnetic NPs	Neglected necrotic–viable region concept and NPs degradation due to nano-bio interaction

### Research gaps and contributions

Despite remarkable progress, challenges remain in modeling tumor fluid dynamics and nanodrug delivery. Existing models use simplifying assumptions, limiting their ability to capture tumor complexity and nano-bio interactions. A comprehensive model accounting for tumor heterogeneity and NP behavior is still needed. After a thorough review of the aforementioned literature, several gaps have been identified, highlighting the need for further investigation. For instance, studies by Hosseinpour et al. [19], Suleman and Riaz [35,36], and Sefidgar et al. [31,46] overlooked critical features such as the necrotic–viable region concept, nano-bio reactions, and transvascular exchange of NPs. Although Mahesh et al. [15,38] considered the concept of necrotic and viable regions, their size variations remain unexplored. Foundational works by Baxter and Jain [7–9], Kashkooli et al. [13], and all the FES literature presented in Table 1 did not account for the complete lymphatic dynamics in surrounding healthy tissues. Additionally, Sefidgar et al. [31] and others [13,30,33,47] studied intravenous nanodrug delivery during hyperthermia but faced challenges with high IFP, which limits NP penetration into deep tumor regions like the necrotic core, reducing effectiveness. This highlights the need for intratumoral injection. Also, it has been observed that the majority of studies have focused on the distribution of a single type of NP, leaving a gap in understanding the comparative behavior of different particle types. Furthermore, a sensitivity test of NP degradation rate constants on their diffusion transport remains underexplored [35,48–50], particularly the role of TAMs and other physiological uptake processes in NP degradation [45].

To address these gaps, this study:

• Incorporates necrotic cores with varying size, viable tumor regions, healthy surrounding tissues, and transvascular exchange mechanisms into the computational framework to better replicate in vivo tumor architecture.
• Incorporates complete functional lymphatic dynamics with existing FES models [15,19,38] and fluid flow resistance parameter to assess their impact on IFP and IFV.
• Evaluates the role of intratumoral injection with varying NP sizes in reducing elevated IFP, enhancing nanodrug distribution and the effectiveness of hyperthermia treatments.
• Simulates and compares multiple nanodrug carrier, including dextran, liposomal, PEG-coated gold, and magnetic particles, alongside conventional doxorubicin, offering a broader understanding of material functionalities.
• Investigates the correlation between NP degradation rate constant and diffusion properties, providing insights to optimize nanodrug delivery through tailored nano-engineering strategies.

### Novelty of the study

This study introduces several novel contributions to the field of tumor drug delivery:

• New insights into interstitial fluid flow dynamics are revealed through the integration of functional lymphatic dynamics.
• Evaluates the critical necrotic radius ($R_{\mathrm{CN}}$) as a key parameter in nanodrug delivery, marking the point where maximum IFP begins to decline and highlighting its role in optimizing treatment strategies for heterogeneous tumors.
• Comparative study on multiple nanodrug carrier alongside conventional agents like doxorubicin broadens the understanding of material characteristics.
• Identifies the NP size range 15to30nm as optimal for therapeutic efficacy and concentration-based hyperthermia applications, providing a clear target for NP design.
• The sensitivity test on degradation rate constant and diffusion parameter of NPs provides insights for tailored nano-engineering strategies.
• Proposed FES reduces mesh size to ~33,000 elements, substantially lowering computational costs compared to previous studies (over 360,000 elements) [15,38] while maintaining accuracy.

## Materials and Methods

### Mathematical model

#### Computational domain

A 2D axisymmetric solid tumor geometry is considered from a 3D spherical tumor [15,38] as computational domain, commonly observed in clinical experiments for relatively small tumors [7,8]. This approach simplifies the physical model while retaining key characteristics of spherical tumor architecture. However, it is acknowledged that this assumption may not fully capture the diversity of tumor morphologies [51], which can vary substantially in shape, size, type, location, and growth patterns [52,53]. Such variations can influence the TME and its response to treatment. For instance, irregularly shaped tumors exhibit distinct fluid flow and solute (NP-based therapeutic drugs or their carriers) transport characteristics compared to spherical tumors [46]. While this simplification is beneficial for isolating and understanding key physical mechanisms, it may limit the direct applicability of findings to irregularly shaped or larger tumors. A more detailed analysis incorporating irregular tumor geometries and integrating patient-specific imaging data, such as magnetic resonance imaging (MRI) or computed tomography (CT) scans, would be a valuable extension of this work in future studies.

**Fig. 1.**
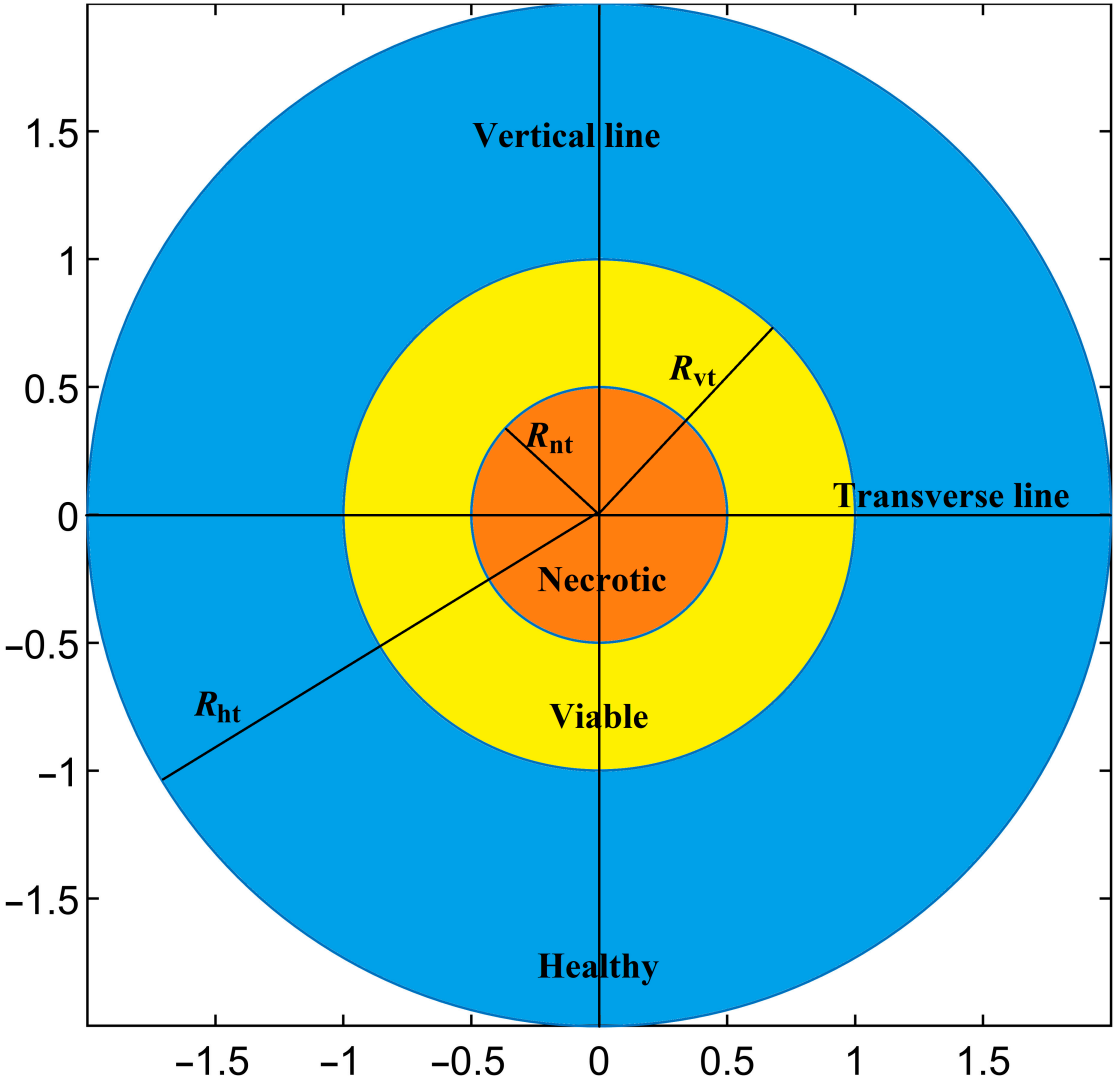
Schematic representation of computational domain.

Here, in this investigation, the 2D domain is divided into 3 distinct concentric circular regions to replicate the architectural complexity of in vivo tumor structures: necrotic core $\;\left(R_{\mathrm{nt}}=0.5\;\mathrm{cm}\right)$, viable tumor $\;\left(R_{\mathrm{vt}}=1\;\mathrm{cm}\right)$, and healthy $\;\left(R_{\mathrm{ht}}=2\;\mathrm{cm}\right)$ region. The schematic computational domain is presented in Fig. 1, providing a visual foundation for the subsequent discussion of findings. The transverse and vertical lines are used to illustrate results along them. A crucial element in tumor modeling is the vasculature. Tumor vasculature, which exhibits a heterogeneous distribution of vessel sizes and shapes, is simplified in this study by using the vascular density distribution S/V, representing the surface area of blood vessels (S) per unit volume of tumor (V). Here, the study assumes zero vascular density in the necrotic core, while in viable and normal tissues, the vascular density is considered homogeneous, maintaining a constant S/V value.

#### Interstitial fluid flow

The conservation of mass for a steady incompressible fluid flow without any mass source and sink in porous medium is given by [15].

$$\nabla \cdot \left(v_{\text{IS}}\right)=0$$ (1)

where $v_{\text{IS}}$ (cm/s) is the IFV, but for a complete vascularized tumor, blood vasculature represents as fluid source and lymphatic system as sink [39]. Therefore, continuity equation for a vascularized tumor is as follows [13]:

$$\nabla \cdot \left(v_{\text{IS}}\right)=\varphi_{\mathrm{VS}}-\varphi_{\mathrm{LS}}$$ (2)

where $\varphi_{\mathrm{VS}}$ and $\varphi_{\mathrm{LS}}$ ($s^{-1}$) are blood vasculature extravasation and lymphatic system loss term, respectively. Consider porous medium to simulate flow within the vascularized tumor surrounded by healthy tissues. The necrotic core, which is the dead part of the tumor, does not have any channels to transport the fluid, so there are no sources or sink there. In the viable tumor zone, microvessels facilitate fluid transmission, yet the absence of a functional lymphatic system restricts drainage [6]. Conversely, normal tissues engage in fluid exchange through both vascular extravasation and lymphatic drainage. Consequently, the mass conservation equation for the tumor, distinguished by its distinct regions, emerges as follows:

∇·vIS=0r<RntφVSRnt≤r≤RvtφVS−φLSr>Rvt (3)

Given the analogous features shared between the interstitium and porous media, the momentum equation is aptly streamlined to conform to Darcy’s law [38]. Consequently, the correlation between IFV and IFP ($P_{\text{IS}}$) is given through the utilization of the following Eq. 4**,** i.e., fluid velocity is directly proportional to the gradient of interstitial pressure.

$$v_{\text{IS}}=-K\nabla P_{\text{IS}}$$ (4)

where K interstitial tissue hydraulic conductivity. Therefore, using Eqs. 3 and 4, we have

−∇·K∇PIS=0forr<RntφVSforRnt≤r≤RvtφVS−φLSforr>Rvt (5)

The fluid source term represents the flow flux out of the vascular wall per unit volume and is governed by Starling’s law [54]. Similarly, the lymphatic drainage term is modeled like blood vessel flow, but without considering the osmotic pressure difference.

$$\varphi_{\mathrm{VS}}=\frac{L_{P}S}{V}\left(P_{V}-P_{\text{IS}}-\sigma_{s}\left(\pi_{V}-\pi_{\text{IS}}\right)\right)$$ (6)

$$\varphi_{\mathrm{LS}}=\frac{L_{\mathrm{PL}}S_{L}}{V}\left(P_{\text{IS}}-P_{L}\right)$$ (7)

Here, $\varphi_{\mathrm{VS}}$ represents the volumetric flow rate out of blood vessels per unit volume of the tumor, while $\varphi_{\mathrm{LS}}$ denotes the volumetric flow rate into lymphatic vessels per unit volume of the tumor. $L_{P}$ is the vascular wall hydraulic conductivity, $\frac{S}{V}$ is the blood vascular density, $P_{V}$ is the microvascular pressure, $\sigma_{s}$ is the osmotic reflection coefficient, $\pi_{V}$ is the capillary oncotic pressure, $\pi_{\text{IS}}$ is the interstitial space oncotic pressure, $\frac{S_{L}}{V}$ is the lymphatic vessels density, $\frac{L_{\mathrm{PL}}S_{L}}{V}$ is the lymphatic filtration coefficient, and $P_{L}$ is the lymphatic hydrostatic pressure.

Here, in the necrotic region $\;r<R_{\mathrm{nt}}$, $\;\varphi_{\mathrm{VS}}=\varphi_{\mathrm{LS}}=0$, as in dead tissue, no fluid exchange through blood vasculature or lymphatic vessels; thus, there are no source and sink terms in this region. Therefore, using Eqs. 5 to 7, IFP can be expressed as:

$$\nabla^{2}P_{\text{IS}}=0\;\text{for}\;r<R_{\mathrm{nt}}$$ (8)

$$-K\nabla^{2}P_{\text{IS}}=\frac{L_{P}S}{V}\left(P_{V}-P_{\text{IS}}-\sigma_{s}\left(\pi_{V}-\pi_{\text{IS}}\right)\right)\;\text{for}\;R_{\mathrm{nt}}\leq r\leq R_{\mathrm{vt}}$$ (9)

$$\begin{array}{c} -K\nabla^{2}P_{\text{IS}}=\frac{L_{P}S}{V}\left(P_{V}-P_{\text{IS}}-\sigma_{s}\left(\pi_{V}-\pi_{\text{IS}}\right)\right)\\ -\frac{L_{\mathrm{PL}}S_{L}}{V}\left(P_{\text{IS}}-P_{L}\right)\;\text{for}\;r>R_{\mathrm{vt}} \end{array}$$ (10)

Rearranging Eq. 10, it can be written as

$$\nabla^{2}P_{\text{IS}}=\frac{\alpha^{2}}{R^{2}}\left(P_{\text{IS}}-P_{\mathrm{SS}}\right)$$ (11)

where we call α as the tumor fluid flow resistance parameter, a dimensionless parameter, which is the ratio of the interstitial resistance to vascular resistance, given as:

$$\alpha =\frac{\text{Interstitial Resistance}}{\text{Vascular Resistance}}=R\sqrt{\frac{\left(L_{P}S+L_{\mathrm{PL}}S_{L}\right)}{\mathrm{KV}}}$$ (12)

For the dead necrotic region, α=0, as there is absence of vascular fluid flow exchange, and for the viable tumor region, $\alpha =R\sqrt{\frac{L_{P}S}{\mathrm{KV}}}$, as there is no lymphatic fluid exchange. The state of equilibrium, denoted as the steady-state pressure $\;P_{\mathrm{SS}}$, is a weighted average of effective pressure ($P_{\mathrm{EF}}$) and $\;P_{L}$, and signifies the interstitial pressure at which the outward flow from the vasculature balances the inward influx into the lymphatics, as shown by Eq. 13. It is important to note that $P_{\mathrm{SS}}$ in the tumor appears to depend on the vascular density S/V and $\;S_{L}/V$. However, since both S/V and $\;S_{L}/V$ are zero in the necrotic core and having constant homogeneous distribution in other tumor regions, $P_{\mathrm{SS}}$ simplifies to a piecewise constant distribution within the tumor, as shown in Eq. 13.1. The effective pressure $P_{\mathrm{EF}}$ defined by the interplay of vascular pressure, plasma osmotic pressure, and interstitial osmotic pressure is given in Eq. 14.

$$P_{\mathrm{SS}}=\frac{L_{P}\left(\frac{S}{V}\right)P_{\mathrm{EF}}+L_{\mathrm{PL}}\left(\frac{S_{L}}{V}\right)P_{L}}{L_{P}\left(\frac{S}{V}\right)+L_{\mathrm{PL}}\left(\frac{S_{L}}{V}\right)}$$ (13)

PSS=PSS_necrotic=0forr<RntPSS_viable=LP_viableSVviablePEF_viableLP_viableSVviableforRnt≤r≤RvtPSS_healthy=LP_healthySVhealthyPEF_healthy+LPLSLVPLLP_healthySVhealthy+LPLSLVforr>Rvt (13.1)

$$P_{\mathrm{EF}}=P_{V}-\sigma_{s}\left(\pi_{V}-\pi_{\text{IS}}\right)$$ (14)

#### Solute transport

The solute flux in the interstitium is described by [34], $\;\vec{N}=-D_{\mathrm{EF}}\nabla C_{\text{IS}}+v_{\text{IS}}C_{\text{IS}}$, where $C_{\text{IS}}$ is the solute concentration in interstitial space, $D_{\mathrm{EF}}$ is the effective diffusion coefficient, and $-D_{\mathrm{EF}}\nabla C_{\text{IS}}$ and $v_{\text{IS}}C_{\text{IS}}$ represent the diffusion and convection terms, respectively. Diffusion and convection are governed by concentration gradient and IFV, respectively. Additionally, the model accounts for solute sources from blood vessels, drainage by lymphatic vessels, and the degradation of solute concentration in the interstitium due to nano-bio chemical reactions within the interstitial space. Consequently, the solute particle delivery equation is conveyed as [34]:

$$\frac{\partial C_{\text{IS}}}{\partial t}+\nabla \cdot \vec{N}=\Phi_{\mathrm{BV}}-\Phi_{\mathrm{LV}}-\Phi_{\mathrm{CL}}$$ (15)

where $\Phi_{\mathrm{BV}}$ and $\Phi_{\mathrm{LV}}$ denote the source and sink terms attributed to transvascular exchange facilitated by blood and lymphatic vessels, respectively. Additionally, $\Phi_{\mathrm{CL}}$ represents the degradation of NP concentration. By applying Darcy’s law in Eq. 4, the velocity of interstitial fluid is determined and subsequently utilized in the transient convection–diffusion–reaction (CDR) equation to model solute transport. Hence, the guiding mathematical formulation to model the dynamics of solute transport is given by the CDR term [7,8,15,38], presented as:

$$\begin{array}{c} \begin{array}{c} \frac{\partial C_{\text{IS}}}{\partial t}=\overset{\text{Diffusion}}{\overbrace{\nabla \cdot \left(D_{\mathrm{EF}}\nabla C_{\text{IS}}\right)}}-\overset{\text{Convection}}{\overbrace{\nabla \cdot \left(v_{\text{IS}}C_{\text{IS}}\right)}}+\overset{\text{Reaction}}{\overbrace{\overset{\text{Transvascular Exchange}}{\overbrace{\left(\Phi_{\mathrm{BV}}-\Phi_{\mathrm{LV}}\right)}}-\overset{\text{Degradation}}{\overbrace{\Phi_{\mathrm{CL}}}}}} \end{array} \end{array}$$ (16)

$$D_{\mathrm{EF}}=\frac{2\xi }{3-\xi }D_{\mathrm{DIS}}\;$$ (17)

$$D_{\mathrm{DIS}}=\frac{k_{B}T}{6\pi \mu n_{r}}$$ (18)

ΦBV=0forr<RntφVS1−σrcCPL+PSVCPL−CISPpecePpec−1forr≥Rnt (19)

ΦLV=0forr<Rnt0forRnt≤r≤RvtφLSCISforr>Rvt (20)

$$P_{\mathrm{pec}}=\frac{\varphi_{\mathrm{VS}}\left(1-\sigma_{\mathrm{rc}}\right)}{P\frac{S}{V}}$$ (21)

$$\Phi_{\mathrm{CL}}=\tau_{\deg}C_{\text{IS}}$$ (22)

As porous medium, the effective diffusion coefficient $D_{\mathrm{EF}}$ governing the NP movement through the interstitial space is modeled by considering the diffusivity in the interstitial fluid $D_{\mathrm{DIS}}$ [55] and the interstitium void fraction ξ within the tumor tissue matrix; $k_{B}$ is Boltzmann constant, T is absolute temperature, μ is the viscosity of interstitial fluid, and $n_{r}$ is NP radius. Additionally, $\Phi_{\mathrm{CL}}$ represents the NP degradation term due to nano-bio interactions influenced by the presence of TAMs and other physiological uptake, $\tau_{\deg}$ stands for the degradation rate constant characterizing the reduction of nanodrug within the extracellular space, P is the vascular permeability coefficient, $\sigma_{\mathrm{rc}}$ is the solute filtration reflection coefficient, $P_{\mathrm{pec}}$ is the Peclet number, and $C_{\mathrm{PL}}$ is plasma concentration. Given the tumor’s relatively small size in comparison to the entire body and blood volume, as well as the slow diffusion velocity within interstitial spaces, the solute injected intratumorally requires considerable time to navigate the complex network and reach the vascular system. Consequently, the resulting effective plasma concentration $C_{\mathrm{PL}}$ may be considered negligible due to the long duration required to achieve meaningful levels. Hence, with this hypothesis, Eq. 19 reduces as:

ΦBV=0forr<Rnt−PSVCISPpecePpec−1forr≥Rnt (23)

The study considered a symmetrical in vivo tumor architecture. Given the symmetry of the geometry, the IFP and solute concentration at the tumor’s center are treated as uniform when addressing fluid flow dynamics and solute transport. The continuity of both the variables and their gradients is maintained across the subdomain boundaries between different regions. A zero-flux Neumann boundary condition is imposed at the outer edge of the healthy tissue, as given in Eqs. 24 and 25.

$$\vec{n}\cdot \left(K\nabla P_{\mathrm{IS}}\right)=0$$ (24)

$$\vec{n}\cdot \left(\nabla C_{\text{IS}}\right)=0$$ (25)

### FEM solution and simulation

Physical laws governing space- and time-dependent phenomena are often represented by partial differential equations (PDEs). In most cases, especially for complex geometries like solid tumors, analytical solutions to these PDEs are rarely feasible, necessitating numerical approximations. One common approach for generating such approximations is the FEM [44]. The FEM, widely used (see Table 1), is a numerical approach that discretizes the problem domain into smaller elements, each of which is controlled by element equations that are taken from the original problem. After that, these separate element equations are combined together to form a global system, which makes the ultimate calculation possible. FEM’s adaptability in tolerating complex geometries has contributed to its increasing popularity.

In the current study, the computational tumor domain (Ω) surrounded by healthy tissue, is modeled and meshed using MATLAB software [56]. The governing fluid flow and solute transport equations along with the specified boundary conditions within bounded 2D or 3D domains are solved using the Galerkin FEM-based algorithm. The process begins with mesh generation, where the complex geometry is discretized into interconnected subdomains. For 2-D problems, the domain Ω is approximated by linear/quadratic triangular elements, forming a mesh with nodes located at the vertices and edges of these triangles. The next step converts the governing equations from its strong form to its weak form, which is then applied to each subdomain, enabling a more efficient and convenient numerical solution.

In order to establish the weak formulation of the original strong form of the governing equations, we consider the governing fluid flow Eq. 11 to assess the pressure variable, Darcy’s Eq. 4 for evaluating velocity, and the solute transport Eq. 16 to analyze the time- and space-dependent concentration of nanodrugs. First and foremost, in order to align with the MATLAB FEM algorithm environment, we first reformulated the fluid and solute transport equations into their scalar forms, as presented in Eqs. 26–29.

$$-\nabla^{2}P_{\text{IS}}+\frac{\alpha^{2}}{R^{2}}P_{\text{IS}}=\frac{\alpha^{2}}{R^{2}}P_{\mathrm{SS}}$$ (26)

$$U_{\text{IS}}=-K\frac{\partial P_{\text{IS}}}{\partial x}$$ (27)

$$V_{\text{IS}}=-K\frac{\partial P_{\text{IS}}}{\partial y}$$ (28)

$$\begin{array}{c} \begin{array}{c} \frac{\partial C_{\text{IS}}}{\partial t}-D_{\mathrm{EF}}\nabla^{2}C_{\text{IS}}+\left(\frac{\partial U_{\text{IS}}}{\partial x}+\frac{\partial V_{\text{IS}}}{\partial y}+P\frac{S}{V}\frac{P_{\mathrm{pec}}}{e^{P_{\mathrm{pec}}}-1}+\varphi_{\mathrm{LS}}+\tau_{\deg}\right)\\ C_{\text{IS}}=-\left(U_{\text{IS}}\frac{\partial C_{\text{IS}}}{\partial x}+V_{\text{IS}}\frac{\partial C_{\text{IS}}}{\partial y}\right) \end{array} \end{array}$$ (29)

where we have considered IFV and IFP gradient vector in Eq. 4 as $\;v_{\text{IS}}=\left(U_{\text{IS}},V_{\text{IS}}\;\right)$ and $\nabla P_{\text{IS}}=\left(\frac{\partial P_{\text{IS}}}{\partial x},\frac{\partial P_{\text{IS}}}{\partial y}\right)$, respectively. In the solute transport Eq. 16, we have $\begin{array}{c} \nabla \cdot \left(v_{\text{IS}}C_{\text{IS}}\right)=\left(\frac{\partial C_{\text{IS}}}{\partial x}\cdot \right. \end{array}$$\begin{array}{c} \left.U_{\text{IS}}+C_{\text{IS}}\cdot \frac{\partial U_{\text{IS}}}{\partial x}\right)+\left(\frac{\partial C_{\text{IS}}}{\partial y}\cdot V_{\text{IS}}+C_{\text{IS}}\cdot \frac{\partial V_{\text{IS}}}{\partial y}\right) \end{array}$. Equations 26 to 29 can be simplified by applying the relevant assumptions and hypotheses for each distinct region of the tumor domain. For example, in the necrotic region, both S/V and $S_{L}/V$ are zero due to the lack of vasculature and lymphatic sinks. In contrast, within the viable region, $S_{L}/V$ is zero because no lymphatics are present. Additionally, $K_{\text{necrotic}},K_{\text{viable}},\text{and}\;K_{\text{healthy}}$represent the hydraulic conductivity of the interstitium in the necrotic, viable, and healthy regions, respectively, Thus, Eqs. 26 to 29 represent the final strong form [57] of the governing equations of the model on the domain Ω, with the boundary condition specified by Eqs. 24 and 25 on $\;{\partial \Omega }_{N}$, the outer edge of the tumor. In general, the boundary of the domain Ω is given by the combination of its Dirichlet and Neumann boundary, i.e., $\partial \Omega ={\partial \Omega }_{D}\cup {\partial \Omega }_{N}$.

Now, the residual/error function of the governing equations is given by:

$$R_{1}=-\nabla^{2}P_{\text{IS}}+\frac{\alpha^{2}}{R^{2}}P_{\text{IS}}-\left(\frac{\alpha^{2}}{R^{2}}P_{\mathrm{SS}}\right)$$ (30)

$$R_{2}=U_{\text{IS}}-\left(-K\frac{\partial P_{\text{IS}}}{\partial x}\right)$$ (31)

$$R_{3}=V_{\text{IS}}-\left(-K\frac{\partial P_{\text{IS}}}{\partial y}\right)$$ (32)

$$\begin{array}{c} R_{4}=\frac{\partial C_{\text{IS}}}{\partial t}-D_{\mathrm{EF}}\nabla^{2}C_{\text{IS}}+\left(\frac{\partial U_{\text{IS}}}{\partial x}+\frac{\partial V_{\text{IS}}}{\partial y}+P\frac{S}{V}\right.\\ \left.\frac{P_{\mathrm{pec}}}{e^{P_{\mathrm{pec}}}-1}+\varphi_{\mathrm{LS}}+\tau_{\deg}\right)C_{\text{IS}}+\left(U_{\text{IS}}\frac{\partial C_{\text{IS}}}{\partial x}+V_{\text{IS}}\frac{\partial C_{\text{IS}}}{\partial y}\right) \end{array}$$ (33)

Now, the first and important step in FEM involves transforming the strong form of the governing equations into their weak (integral) form [57]. This is achieved by multiplying the governing equations by the test functions $\;u_{h}$,$\;v_{h}$, $w_{h}$ and $q_{h}$, and integrating over the domain Ω. The key idea is to take the residuals $\;R_{i}$’s (i=1,2,3,4) of the governing equations and project them onto the subspaces defined by the test functions using the inner products. By setting the resulting integrals to zero, these projections minimize the residual errors, enabling optimal numerical approximations for solving the governing equations.

$$\begin{array}{c} \left(R_{1},\;u_{h}\right)=\int_{\Omega }\left(-\nabla^{2}P_{\text{IS}}+\frac{\alpha^{2}}{R^{2}}P_{\text{IS}}-\frac{\alpha^{2}}{R^{2}}P_{\mathrm{SS}}\right)\cdot u_{h}d\Omega =0 \end{array}$$ (34)

$$\begin{array}{c} \left(R_{2},v_{h}\right)=\int_{\Omega }\left(U_{\text{IS}}+K\frac{\partial P_{\text{IS}}}{\partial x}\right)\cdot v_{h}d\Omega =0 \end{array}$$ (35)

$$\begin{array}{c} \left(R_{3},w_{h}\right)=\int_{\Omega }\left(V_{\text{IS}}+K\frac{\partial P_{\text{IS}}}{\partial y}\right)\cdot w_{h}d\Omega =0 \end{array}$$ (36)

$$\begin{array}{c} \begin{array}{c} \left(R_{4},\;q_{h}\right)=\int_{\Omega }\left(\frac{\partial C_{\text{IS}}}{\partial t}-D_{\mathrm{EF}}\nabla^{2}C_{\text{IS}}+\left(\frac{\partial U_{\text{IS}}}{\partial x}+,\frac{\partial V_{\text{IS}}}{\partial y},+,P,\frac{S}{V}\right.\right.\\ \left.\left.\frac{P_{\mathrm{pec}}}{e^{P_{\mathrm{pec}}}-1}+\varphi_{\mathrm{LS}}+\tau_{\deg}\right)C_{\text{IS}}+\left(U_{\text{IS}}\frac{\partial C_{\text{IS}}}{\partial x}+V_{\text{IS}}\frac{\partial C_{\text{IS}}}{\partial y}\right)\right)\cdot q_{h}d\Omega =0 \end{array} \end{array}$$ (37)

The test functions $u_{h}$,$\;v_{h}$, $w_{h},$ and $q_{h}$ are drawn from the functional spaces and satisfy the condition of being zero on the Dirichlet boundary, meaning $u_{h}=0$,$\;v_{h}=0$, $w_{h}=0$, and $q_{h}=0$, on ${\partial \Omega }_{D}$ (if applicable). This approach can be understood as applying a weighted residual method, where the test functions act as the weighting factors. The admissible solutions $P_{\text{IS}},U_{\text{IS}},V_{\text{IS}}$, and $C_{\text{IS}}$ for the weak forms are chosen to meet the Dirichlet boundary conditions on $\;{\partial \Omega }_{D}$.

Integrating by parts the second-order Laplace term of Eqs. 26 to 29 using Green’s identity formula [58], we have:

$$\begin{array}{c} \begin{array}{c} \int_{\Omega }\left(\nabla P_{\text{IS}}\nabla u_{h}\right)d\Omega -\int_{{\partial \Omega }_{N}}\vec{n}\cdot \left(\nabla P_{\text{IS}}\right)u_{h}d{\partial \Omega }_{N}\\ +\int_{{\partial \Omega }_{D}}\vec{n}\cdot \left(\nabla P_{\text{IS}}\right)u_{h}d{\partial \Omega }_{D}+\int_{\Omega }\;\frac{\alpha^{2}}{R^{2}}P_{\text{IS}}u_{h}d\Omega -\int_{\Omega }\frac{\alpha^{2}}{R^{2}}P_{\mathrm{SS}}u_{h}d\Omega =0 \end{array} \end{array}$$ (38)

$$\int_{\Omega }U_{\text{IS}}v_{h}d\Omega +\int_{\Omega }K\frac{\partial P_{\text{IS}}}{\partial x}v_{h}d\Omega =0$$ (39)

$$\int_{\Omega }V_{\text{IS}}w_{h}d\Omega +\int_{\Omega }K\frac{\partial P_{\text{IS}}}{\partial y}w_{h}d\Omega =0$$ (40)

$$\begin{array}{c} \begin{array}{c} \int_{\Omega }\frac{\partial C_{\text{IS}}}{\partial t}q_{h}d\Omega +\int_{\Omega }\left(D_{\mathrm{EF}}\nabla C_{\text{IS}}\nabla q_{h}\right)d\Omega -\int_{{\partial \Omega }_{N}}\vec{n}\cdot \left(D_{\mathrm{EF}}\nabla C_{\text{IS}}\right)q_{h}d{\partial \Omega }_{N}\\ +\int_{{\partial \Omega }_{D}}\vec{n}\cdot \left(D_{\mathrm{EF}}\nabla C_{\text{IS}}\right)q_{h}d{\partial \Omega }_{N}+\int_{\Omega }\;\left(\frac{\partial U_{\text{IS}}}{\partial x}+\frac{\partial V_{\text{IS}}}{\partial y}+P\frac{S}{V}\frac{P_{\mathrm{pec}}}{e^{P_{\mathrm{pec}}}-1}+\varphi_{\mathrm{LS}}+\tau_{\deg}\right)\\ C_{\text{IS}}q_{h}d\Omega \;+\int_{\Omega }\left(U_{\text{IS}}\frac{\partial C_{\text{IS}}}{\partial x}+V_{\text{IS}}\frac{\partial C_{\text{IS}}}{\partial y}\right)q_{h}d\Omega =0 \end{array} \end{array}$$ (41)

Equations 38 to 41 are known as the weak forms of the governing equations. Notably, the second-order derivatives in the original governing Eqs. 26 to 29 are reduced to first-order derivatives in these weak forms, leading to what is termed a “weakened” formulation. As a result, the solutions obtained are numerical approximations, referred to as weak solutions, rather than the exact, strong (analytical) solutions.

Now, applying the boundary conditions given in Eqs. 24 and 25, and nullifying the integral term defined for the Dirichlet boundary, as we do not have any Dirichlet boundary, Eqs. 38 to 41 reduces to:

$$\begin{array}{c} \int_{\Omega }\left(\nabla P_{\text{IS}}\nabla u_{h}\right)d\Omega +\int_{\Omega }\;\frac{\alpha^{2}}{R^{2}}P_{\text{IS}}u_{h}d\Omega =\int_{\Omega }\frac{\alpha^{2}}{R^{2}}P_{\mathrm{SS}}u_{h}d\Omega \end{array}$$ (42)

$$\int_{\Omega }U_{\text{IS}}v_{h}d\Omega =-\int_{\Omega }K\frac{\partial P_{\text{IS}}}{\partial x}v_{h}d\Omega$$ (43)

$$\int_{\Omega }V_{\text{IS}}w_{h}d\Omega =-\int_{\Omega }K\frac{\partial P_{\text{IS}}}{\partial y}w_{h}d\Omega$$ (44)

$$\begin{array}{c} \int_{\Omega }\frac{\partial C_{\text{IS}}}{\partial t}q_{h}d\Omega +\int_{\Omega }\left(D_{\mathrm{EF}}\nabla C_{\text{IS}}\nabla q_{h}\right)d\Omega \\ +\int_{\Omega }\;\left(\frac{\partial U_{\text{IS}}}{\partial x}+\frac{\partial V_{\text{IS}}}{\partial y}+P\frac{S}{V}\frac{P_{\mathrm{pec}}}{e^{P_{\mathrm{pec}}}-1}+\varphi_{\mathrm{LS}}+\tau_{\deg}\right)\\ C_{\text{IS}}q_{h}d\Omega =-\int_{\Omega }\left(U_{\text{IS}}\frac{\partial C_{\text{IS}}}{\partial x}+V_{\text{IS}}\frac{\partial C_{IS}}{\partial y}\right)q_{h}d\Omega \end{array}$$ (45)

**Fig. 2.**
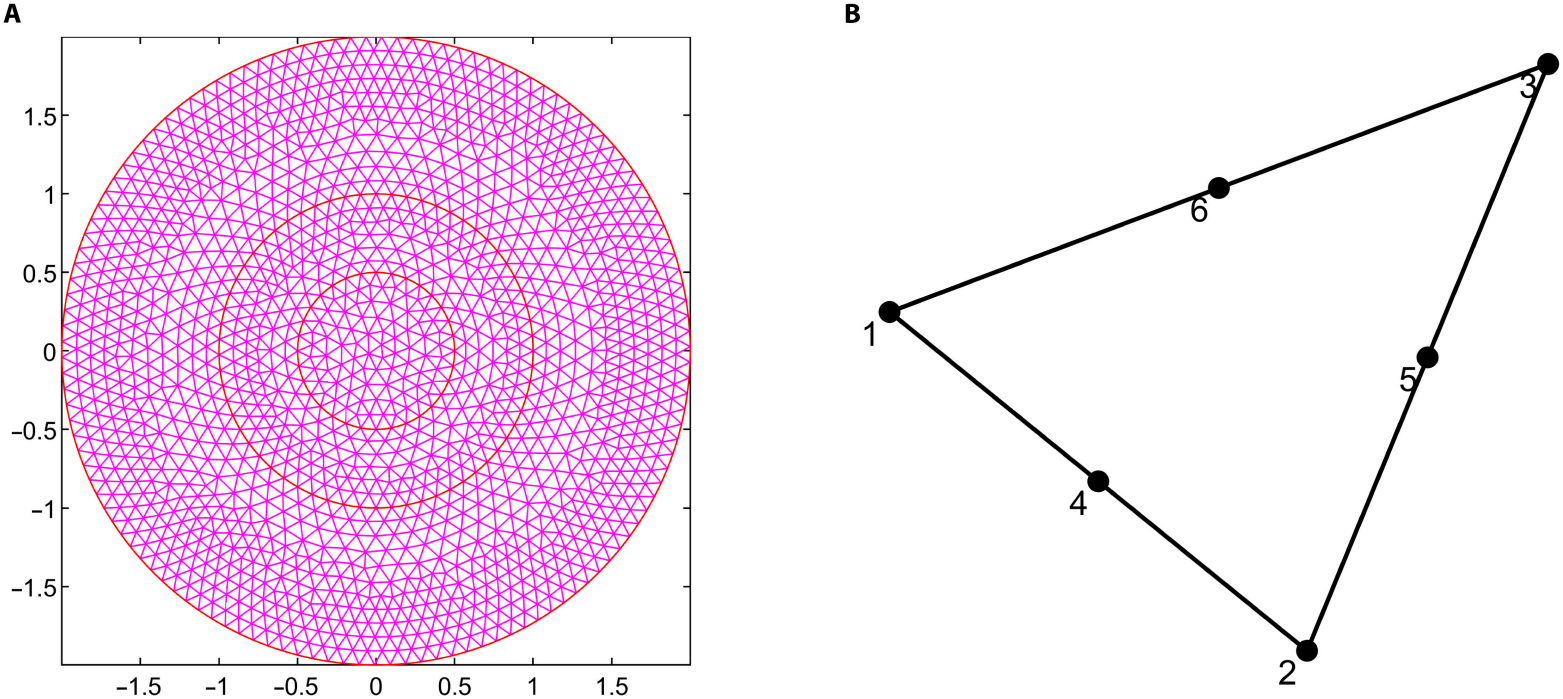
(A) Schematic illustration of the domain Ω, subdivided into smaller quadratic triangular elements $\;\Omega^{e}$. (B) A representation of a quadratic triangular element with 6 nodes: 3 positioned at the vertices and 3 at the midpoints of the edges.

Up to this point, all calculations have been performed over the entire domain Ω, which represents the global domain of the problem. Consequently, the trial and admissible functions span infinite-dimensional functional spaces. Now, the next step is discretizing the weak form, dividing Ω into smaller elements, $\;\Omega^{e}$, where $\Omega =\cup \Omega^{e}$. Essentially, this involves projecting the weak form onto a finite-dimensional subspace. In this study, the domain was discretized using triangular elements with quadratic geometric order (Fig. 2A), providing greater accuracy. In a 2D mesh, these quadratic triangular elements consist of 6 nodes, 3 at the triangle’s corners and 3 at the midpoints of its edges (Fig. 2B), offering higher precision compared to linear order elements.

Let$\;P_{\text{IS}}^{e}$, $U_{\text{IS}}^{e}$, $V_{\text{IS}}^{e}$, and $C_{\text{IS}}^{e}$ represent the finite-dimensional approximations of the permissible solutions, and $u_{h}^{e}$, $v_{h}^{e}$, $w_{h}^{e}$, and $q_{h}^{e}$ denote the corresponding test functions, all defined on the subdomain $\Omega^{e}$. Therefore, the discretized weak form of the governing equation at the element level, can be written as:

$$\begin{array}{c} \begin{array}{c} \int_{\Omega^{e}}\left(\nabla P_{\text{IS}}^{e}\nabla u_{h}^{e}\right)d\Omega^{e}+\int_{\Omega^{e}}\;\frac{\alpha^{2}}{R^{2}}P_{\text{IS}}^{e}u_{h}^{e}d\Omega^{e}=\int_{\Omega^{e}}\frac{\alpha^{2}}{R^{2}}P_{\mathrm{SS}}^{e}u_{h}^{e}d\Omega^{e} \end{array} \end{array}$$ (46)

$$\int_{\Omega^{e}}U_{\text{IS}}^{e}v_{h}^{e}d\Omega^{e}=-\int_{\Omega^{e}}K\frac{\partial P_{\text{IS}}^{e}}{\partial x}v_{h}^{e}d\Omega^{e}$$ (47)

$$\int_{\Omega^{e}}V_{\text{IS}}^{e}w_{h}^{e}d\Omega^{e}=-\int_{\Omega^{e}}K\frac{\partial P_{\text{IS}}^{e}}{\partial y}w_{h}^{e}d\Omega^{e}$$ (48)

$$\begin{array}{c} \int_{\Omega^{e}}\frac{\partial C_{\text{IS}}^{e}}{\partial t}q_{h}^{e}d\Omega^{e}+\int_{\Omega^{e}}\left(D_{\mathrm{EF}}\nabla C_{\text{IS}}^{e}\nabla q_{h}^{e}\right)d\Omega^{e}\\ +\int_{\Omega^{e}}\;\left(\frac{\partial U_{\text{IS}}^{e}}{\partial x}+\frac{\partial V_{\text{IS}}^{e}}{\partial y}+P\frac{S}{V}\frac{P_{\mathrm{pec}}}{e^{P_{\mathrm{pec}}}-1}+\varphi_{\mathrm{LS}}+\tau_{\deg}\right)\\ C_{\text{IS}}^{e}q_{h}^{e}d\Omega^{e}=-\int_{\Omega^{e}}\left(U_{\text{IS}}^{e}\frac{\partial C_{\text{IS}}^{e}}{\partial x}+V_{\text{IS}}^{e}\frac{\partial C_{\text{IS}}^{e}}{\partial y}\right)q_{h}^{e}d\Omega^{e} \end{array}$$ (49)

Let $\psi_{i}^{e}$ (where$\;i=1,2,3,\ldots ,N_{p}$) represent the basis functions for the subspace containing $\;P_{\text{IS}}^{e}$, $U_{\text{IS}}^{e}$, and $V_{\text{IS}}^{e}$, and $u_{h}^{e}$, $v_{h}^{e}$, and $w_{h}^{e}$. Since pressure and velocity are interrelated, approximating one will determine the other. Similarly, $\phi_{i}^{e}\left(x,y\right)$ denotes the basis functions in the spatial domain for the subspace containing $C_{\text{IS}}^{e}$ and $q_{h}^{e}$. These basis functions, commonly referred to as local element trial functions, are also known as interpolation or shape functions. Thus, any of the functions $\;P_{\text{IS}}^{e}$, $U_{\text{IS}}^{e}$, $V_{\text{IS}}^{e}$, or $C_{\text{IS}}^{e}$ can be expressed as a linear combination of these basis functions:

$$P_{\text{IS}}^{e}=\sum_{i=1}^{N_{p}}U_{i}^{e}\psi_{i}^{e}$$ (50)

$$U_{\text{IS}}^{e}=\sum_{j=1}^{N_{p}}V_{j}^{e}\psi_{j}^{e}$$ (51)

$$V_{\text{IS}}^{e}=\sum_{k=1}^{N_{p}}W_{k}^{e}\psi_{k}^{e}$$ (52)

$$C_{\text{IS}}^{e}=\sum_{l=1}^{N_{d}}Z_{l}^{e}\left(t\right)\phi_{l}^{e}\left(x,y\right)$$ (53)

Here, $U_{i}^{e}$, $V_{i}^{e}$, and $W_{i}^{e}$ represent the undetermined scalar coefficients, also known as the nodal values of $P_{\text{IS}}^{e}$, $U_{\text{IS}}^{e}$, and $V_{\text{IS}}^{e}$ at specific local nodes within element $\Omega^{e}$, whereas $Z_{l}^{e}\left(t\right)$ gives the nodal values of $C_{\text{IS}}^{e}$ in the temporal domain. Next, we employ the standard Galerkin method [57] by choosing the test functions $u_{h}^{e}$, $v_{h}^{e}$, $w_{h}^{e}$, and $q_{h}^{e}$ to be identical to their corresponding basis/trial functions, i.e., $u_{h}^{e}=\psi_{m}^{e}$, $v_{h}^{e}=\psi_{n}^{e}$, $w_{h}^{e}=\psi_{p}^{e}$, and $q_{h}^{e}=\phi_{t}^{e}$. Substituting Eqs. 50 to 53 into Eqs. 46 to 49 and performing the integration over the element yields a system of local algebraic finite element equations from the first 3 equations and a system ordinary differential equation (ODE) from the last equation. Specifically, this leads to a system of $N_{p}$ equations with $N_{p}$ unknowns for each $\;U_{i}^{e}$, $V_{j}^{e}$, and $W_{i}^{e}$, which correspond to the pressure and velocity fields. These fields are then used in the transient CDR equation for solute transport, resulting in a system of $N_{d}$ ODEs with $N_{d}$ unknowns for $\;Z_{l}^{e}$.

The standard Galerkin-based FEM formulation [57] yields a local system of algebraic equations at the element level, expressed as $K^{e}U^{e}=F^{e}$ for stationary linear problems. In the case of time-dependent problems, this leads to a set of ODEs: $M^{e}\frac{dV^{e}}{\mathrm{dt}}+N^{e}V^{e}=G^{e}$. The coefficient matrices and the right-hand-side source vector are derived from integrals involving the basis functions and parameter coefficients, which are based on tissue-specific parameters for different regions of the geometry, as detailed in Table 2. The vectors $U^{e}$ and $V^{e}$ represent the solution vectors for the system. The final step involves assembling these local systems into a global system of algebraic equations that models the entire problem. This global system is then solved numerically using a FEM-based solver appropriate to the specific problem.

**Table 2.** Tissue and vasculature physiological parameters value used in the current study

Parameter	Description	Tissue region	Value	Ref.
$R_{\mathrm{nt}}\;\left[\mathrm{cm}\right]$ $R_{\mathrm{vt}}\;\left[\mathrm{cm}\right]$ $R_{\mathrm{ht}}\;\left[\mathrm{cm}\right]$	Tissue radius	Necrotic Viable Healthy	0.5 1 2	–
$L_{p}\;\left[\frac{\mathrm{cm}}{\text{mmHg}\cdot s}\right]$	Microvascular wall hydraulic conductivity	Necrotic Viable Healthy	$2.8\times 10^{-7}$ $2.8\times 10^{-7}$ $3.6\times 10^{-8}$	[8,11,15,31,46]
$K\left[\frac{cm^{2}}{\text{mmHg}\cdot s}\right]$	Interstitial tissue hydraulic conductivity	Necrotic Viable Healthy	$4.13\times 10^{-8}$ $4.13\times 10^{-8}$ $8.53\times 10^{-9}$	
$\frac{S}{V}\;\left[cm^{-1}\right]$	Vascular density	Viable Healthy	200 70	
$\sigma_{s}$	Osmotic reflection coefficient	Viable Healthy	0.82 0.91	
$\pi_{V}\;\left[\text{mmHg}\right]$	Capillary oncotic pressure	Viable Healthy	20 20	
$\pi_{\text{IS}}\;\left[\text{mmHg}\right]$	Interstitial space oncotic pressure	Viable Healthy	15 10	
$P_{V}\;\left[\text{mmHg}\right]$	Microvascular pressure	Viable Healthy	15.6 15.6	
$P\;\left[\mathrm{cm}/s\right]$	Permeability	Viable Healthy	$5.73\times 10^{-9}$ $0.73\times 10^{-9}$	[8,9]
$\frac{L_{\mathrm{PL}}S_{L}}{V}\;\left[\frac{1}{\text{mmHg}\cdot s}\right]$	Lymphatic filtration coefficient	Healthy	$1.1\times 10^{-5}$	
ξ	Interstitium void fraction	Necrotic Viable Healthy	0.6 0.4 0.26	[15,19]
$\sigma_{\mathrm{rc}}$	Solute filtration reflection coefficient	–	0.5	[13]
$\tau_{\deg}\;\left[1/s\right]$	Solute physiological sink/degradation rate	–	$5.8\times 10^{-6}$	[50]
$k_{B}\;\left[J/K\right]$	Boltzmann constant	–	$1.38\times 10^{-23}$	[15]
$T\;\left[K\right]$	Temperature	–	310	[15]
$\mu \;\left[\mathrm{Pa}\cdot s\right]$	Interstitial fluid viscosity	–	$1\times 10^{-3}$	[15,38]

Solving for interstitial fluid flow yields distributions of IFP and the corresponding IFV, while the solute transport (CDR) equation provides nanodrug concentration in the interstitial space (NCIS). In order to ensure convergence of the model solution, residual, absolute, and relative tolerances are set at $\;10^{-4},10^{-6},\text{and}\;10^{-3},$respectively. In order to examine how element size affects numerical results of IFP and IFV, 5 different mesh sizes are tested using quadratic triangular mesh 2D elements. The results across these meshes have been compared to ensure mesh independence, and it is found that results stabilized beyond the third mesh size. Therefore, all simulations are conducted using the optimal element size determined from test case 4 (T4), where the maximum edge length of the mesh, *H*_max_ $\;(L_{\mathrm{ME}}=0.03\;\mathrm{cm})$. This choice is selected over other configurations and ensured smooth and accurate results while minimizing computational cost. The final mesh used in this study consists of 32,939 elements and 66,298 nodes. Full details of the mesh independence study are provided in the subsequent section.

#### Mesh independence study

**Fig. 3.**
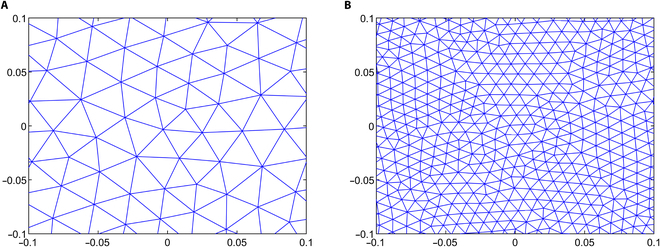
Closer view of the mesh created using mesh parameters of (A) case 4 and (B) case 5.

**Fig. 4.**
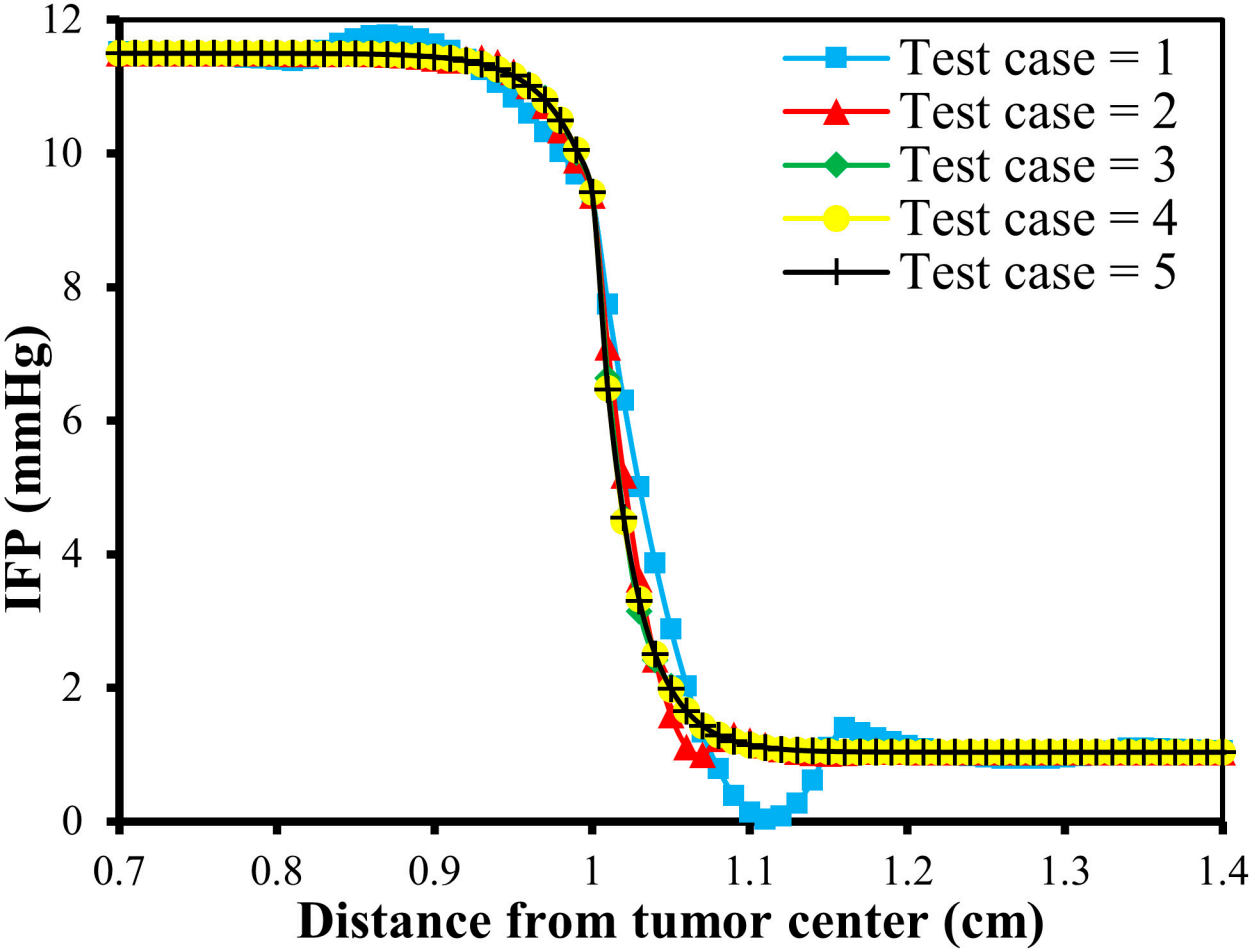
Mesh independence test results for IFP.

**Fig. 5.**
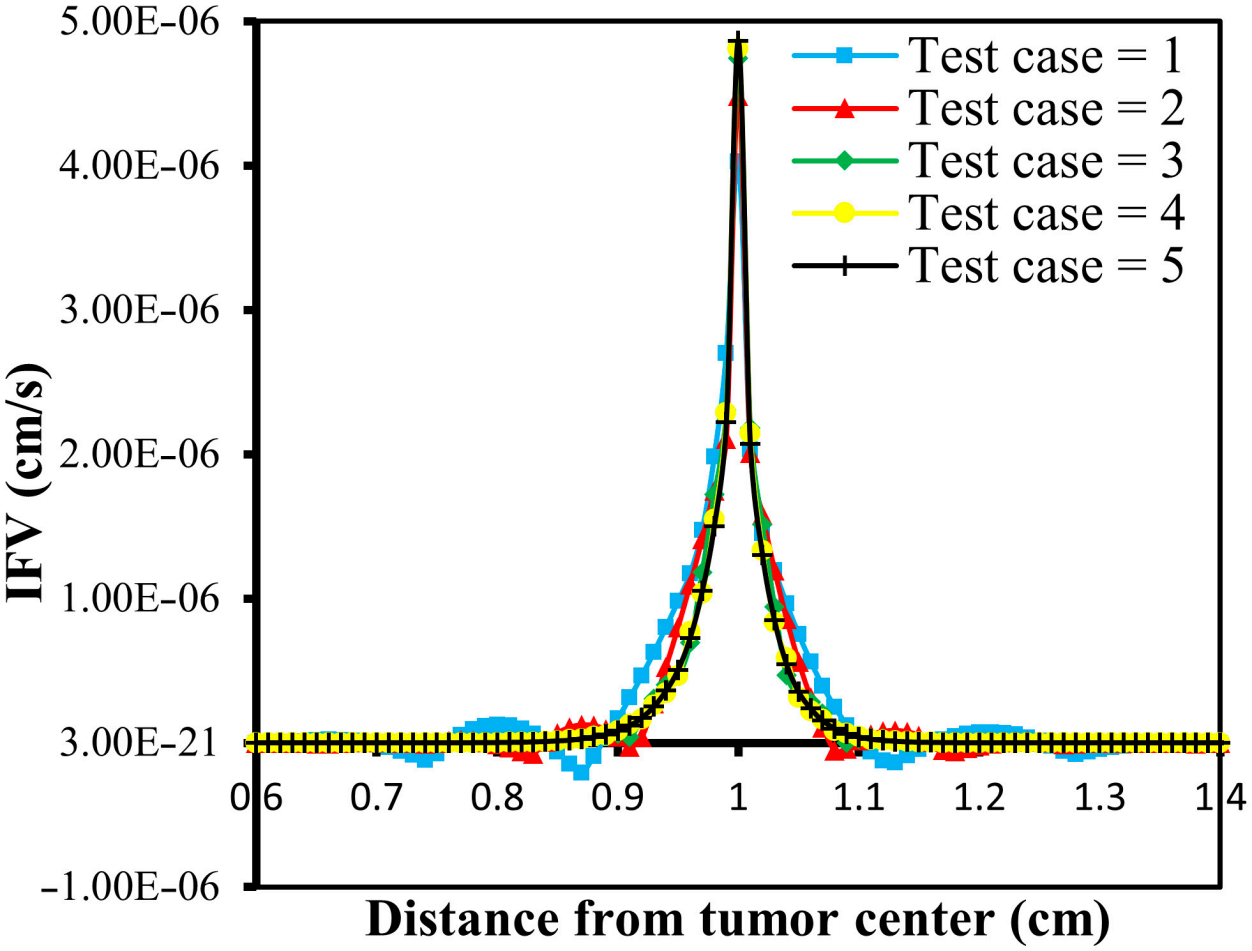
Mesh independence test results for IFV.

Numerical simulation results are generally influenced by the mesh size of the discretized domain. In this study, a mesh independence test is conducted using 5 different mesh sizes, ranging from test case 1 to test case 5, with progressively smaller element sizes. In particular, IFP and IFV profiles are considered to evaluate mesh independence. Detailed information about the generated quadratic triangular mesh elements for all the test cases is given in Table 3. The last column of Table 3 displays the meshes created using the mesh parameters for each test case of the considered geometry. Figure 3A and B provide a closer view of the mesh at the center of the necrotic core for test cases 4 and 5. The results of the mesh independence test are presented in Figs. 4 and 5, which shows the IFP and IFV profiles along the transverse radial line from the tumor center to the outer core boundary, respectively. It can also be observed that after the test case 3, the profiles of IFP and IFV are nearly identical, with the curves almost overlapping. Additionally, while smaller element sizes substantially increase the number of elements, the differences in the profiles remain minimal. Therefore, in order to maintain the mesh independence, the final computational mesh considered in this study includes 32,939 elements and 66,298 nodes (test case 4) (see Table 3).

**Table 3.** Mesh details for IFP and IFV analysis

Mesh test case no.	Maximum element edge length (cm)	Total number of elements (E) and nodes (N)	Mesh figure
1	0.2	E: 770 N: 1,603	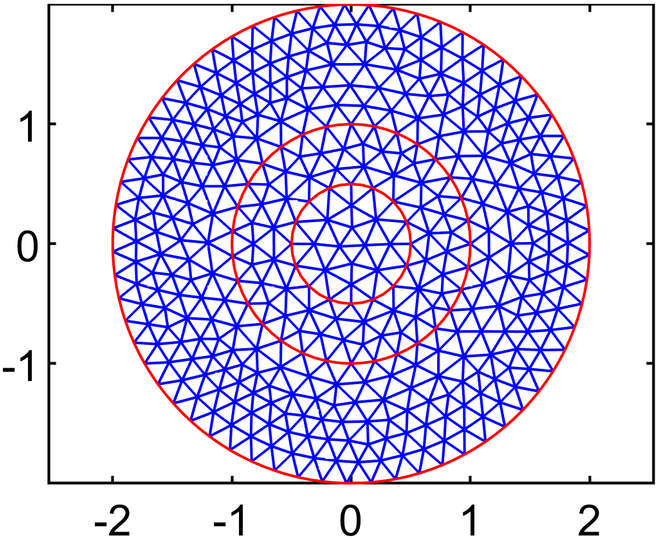
2	0.1	E: 2,886 N: 5,899	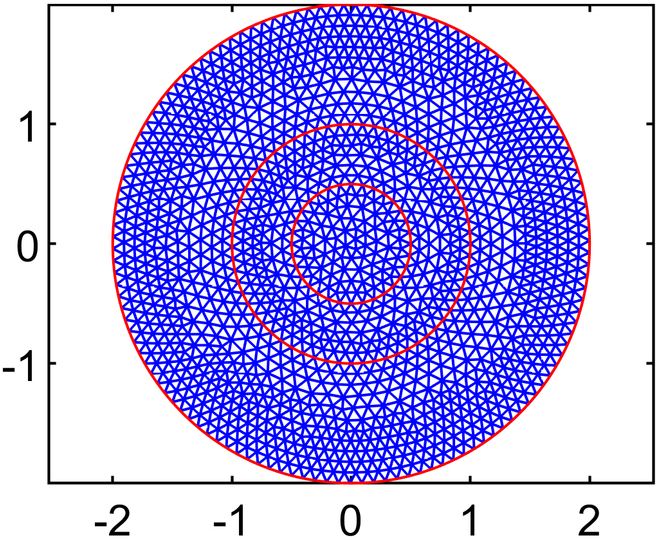
3	0.05	E: 11,713 N: 23,680	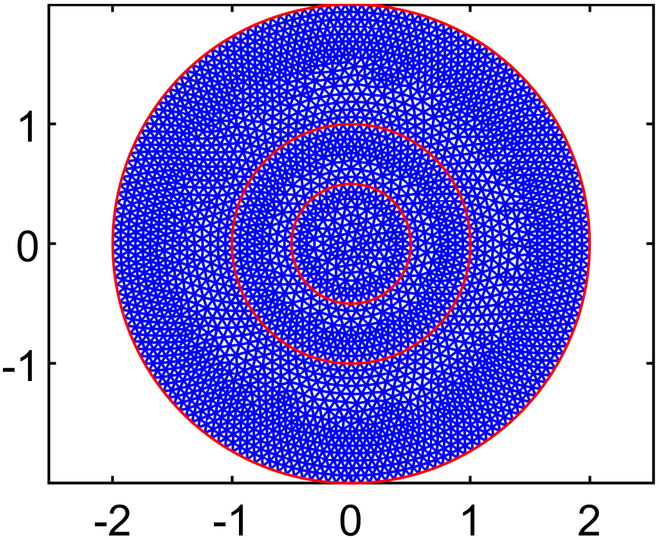
4	0.03	E: 32,939 N: 66,298	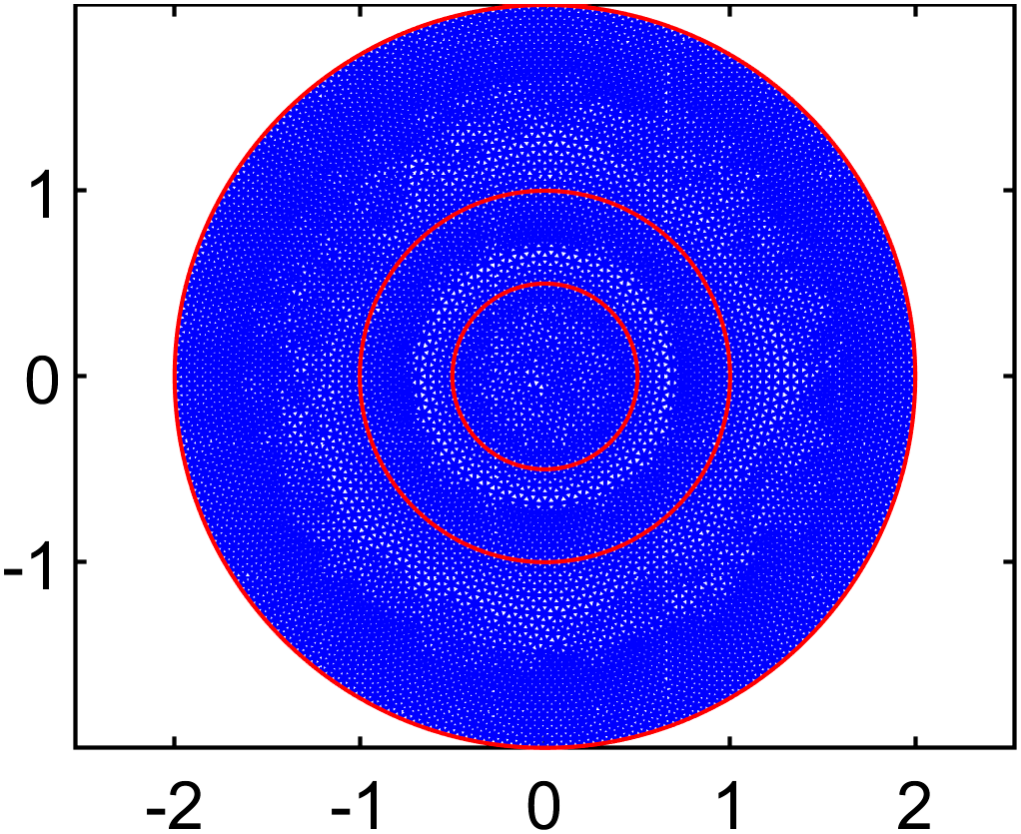
5	0.01	E: 282,271 N: 565,800	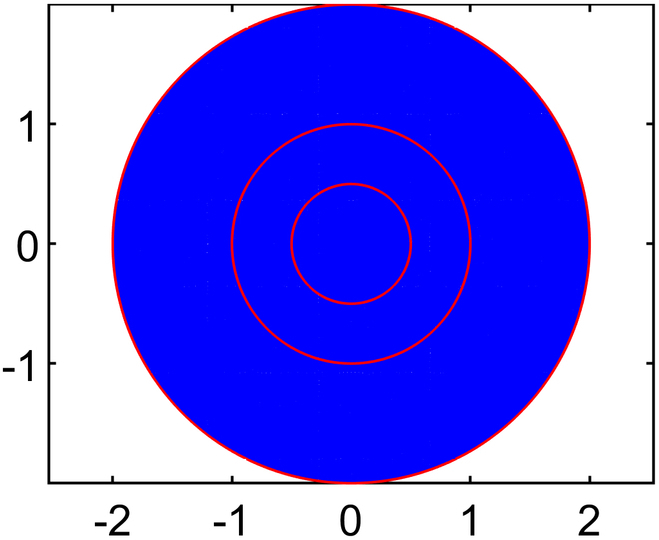

#### Validation

The proposed model is validated through direct comparisons of its computational simulations with established benchmarks from prior experimental and computational studies. This involves simplifying the model and incorporating parameter values from the previous literature to facilitate accurate, one-to-one validation.

**Fig. 6.**
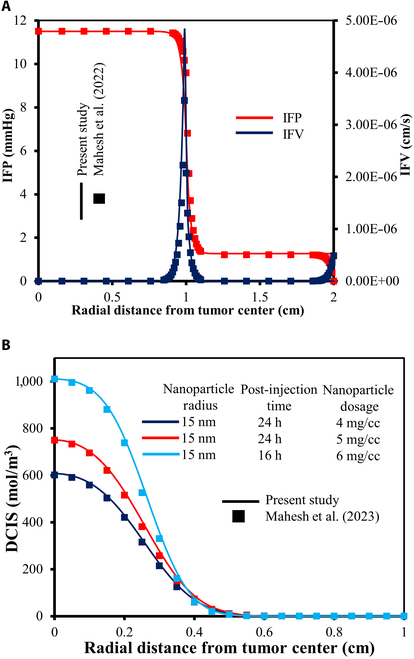
Numerical model validation: (A) IFP and IFV profile. (B) Nanoparticle concentration profiles within the tumor at various post-injection times for the different dosages*.*

The interstitial fluid flow model is validated by comparing its outcomes with those from a study by Mahesh et al. [15], which was validated using theoretical and experimental literature by Baxter and Jain [7]. The model in Mahesh et al.’s work considers a 3D spherical tumor structure, with distinct radial zones: a necrotic core (0.5cm radius), a viable region (1cm radius), and surrounding healthy tissue (2cm radius). The physiological and transport parameters utilized in their model align with the ones specified in Table 2 of this study, except a Dirichlet boundary condition for IFP ($P_{\text{IS}}=0$) is applied at the outermost surface. All other parameters and interface boundary conditions are the same as those used in this study. The governing equations and boundary conditions were solved using the FEM in COMSOL Multiphysics software [59]. All simulations were conducted with a refined mesh comprising 363,692 elements. It is important to note that for the validation scenario described above, the complete functional lymphatic dynamics is neglected ($P_{L}=0$) in order to ensure an exact one-to-one comparison with the reference case. As shown in Fig. 6A, a comparison of IFP and IFV between the 2 models reveals strong consistency, with minor deviations near the outer boundary attributed to differences in the applied boundary conditions.

In order to validate the solute transport model, this study references the numerical work of Mahesh et al. [38], which was validated against the benchmark study by Steuperaert et al. [60]. The computational domain geometry, interface boundary conditions, and other physical parameters were consistent with those used in Mahesh et al.’s earlier work [15]. Fluid flow and solute diffusion parameters in their model match those detailed in Table 2 of this study, with a zero-flux Neumann boundary condition applied at the outer edge, similar to our approach. Their [38] investigation considered 3 NP sizes (15,20,and25nm) and 3 dosages ($4,5,\text{and}\;6\;\mathrm{mg}/cm^{3}$). The injected nanofluid was distributed uniformly within a spherical region with a radius of 3mm after the infusion, achieving a concentration equal to the NP dose used in the prepared nanofluid. Simulations of post-injection solute transport were conducted in COMSOL Multiphysics software [59] using a mesh comprising 666,578 elements. Figure 6B shows the NP concentration profiles within the tumor at various post-injection times for the different dosages considered. The current computational framework results exhibit a strong alignment with the findings.

This confirms the current FES model’s framework accuracy in predicting fluid flow and solute transport dynamics within in vivo tumor structures. A key novelty of our simulation is the reduction of the model to an axisymmetric 2D geometry and its solution using standard Galerkin FEM. This approach utilizes approximately 33,000 elements, considerably fewer than the elements used in the studies by Mahesh et al. [15,38], respectively. This substantially reduces computational costs while preserving accuracy, highlighting its efficiency and precision.

## Results and Discussion

### Fluid flow in the interstitial space

Tumors commonly manifest as a convoluted and impaired network of microvessels, characterized by heightened permeability that results in the seepage of fluid and plasma into the interstitial space [6]. The lymphatic system, tasked with draining surplus fluid and waste materials from tissues, encounters challenges within tumor environments where lymphatic vessels may be compromised or inadequate, impeding efficient fluid removal [39]. This culminates in the accumulation of fluids and other substances within the tumor region, elevating internal pressures. The IFP within both tumor and normal tissues play a pivotal role in drug delivery dynamics, presenting a formidable barrier to effective penetration.

Now, the study explores the effect of varying the tumor fluid flow resistance parameter α on the distribution of IFP and analyzes the impact of VN on this dynamic. Using the baseline parameters from Table 2 and the VN parameters from Table 4, the study conducts simulations for the model equations with the defined boundary conditions. Additionally, the study has compared the results by considering the presence $\left(P_{L}\neq 0\right)$ and absence $\left(P_{L}=0\right)$ of complete lymphatic vessel dynamics in the healthy region of the tumor. In the healthy region, fluid transport is caused by both blood vasculature and lymphatics; hence, the study considers hydrostatic pressure in lymphatic vessel $P_{L}=-4\;$mmHg [39], in order to replicate the case of complete functional lymphatic dynamics.

**Table 4.** Physiological parameters for vascular normalization

VN (%) level	Parameters			
	$K_{t}$	$L_{p}$	$\frac{S}{V}$	$\sigma_{s}$
0% VN (baseline values)	$4.13\times 10^{-8}$	$2.8\times 10^{-7}$	200	0.82
50% VN	$2.49\times 10^{-8}$	$1.585\times 10^{-7}$	135	0.865
100% VN	$8.53\times 10^{-9}$	$3.6\times 10^{-8}$	70	0.91

**Fig. 7.**
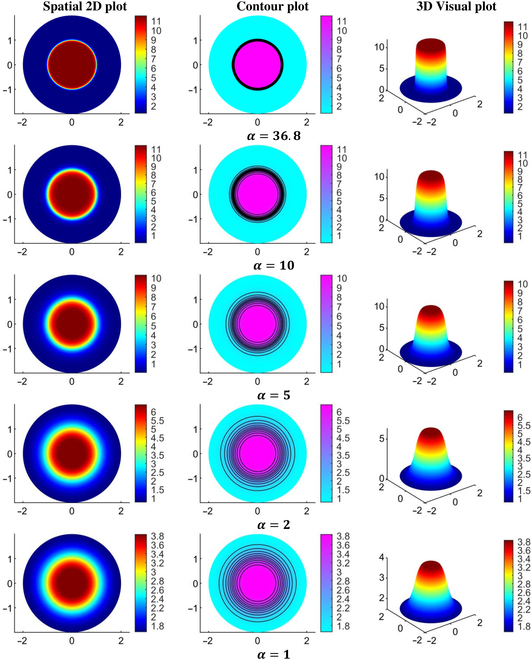
Interstitial fluid pressure (in mmHg) for various α values.

**Fig. 8.**
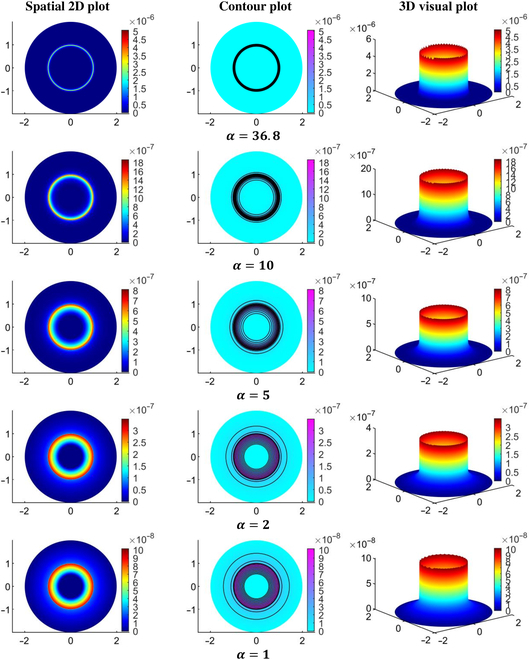
Interstitial fluid velocity (in cm/s) for various α values.

**Fig. 9.**
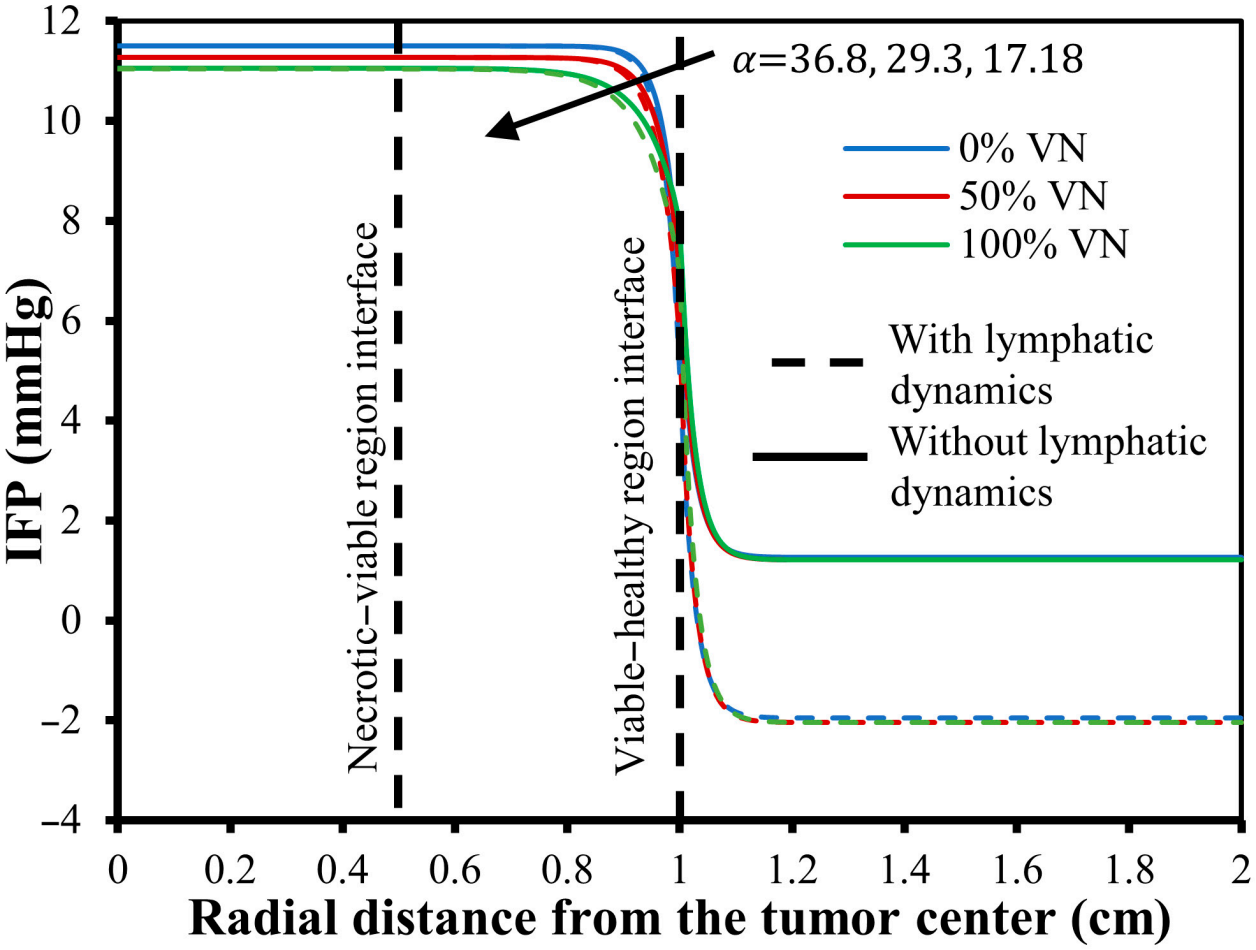
IFP distribution for the baseline parameters given in the table w.r.t. 0%, 50%, and 100% VN with (small dash symbol) and without (solid line) considering functional lymphatics dynamics.

**Fig. 10.**
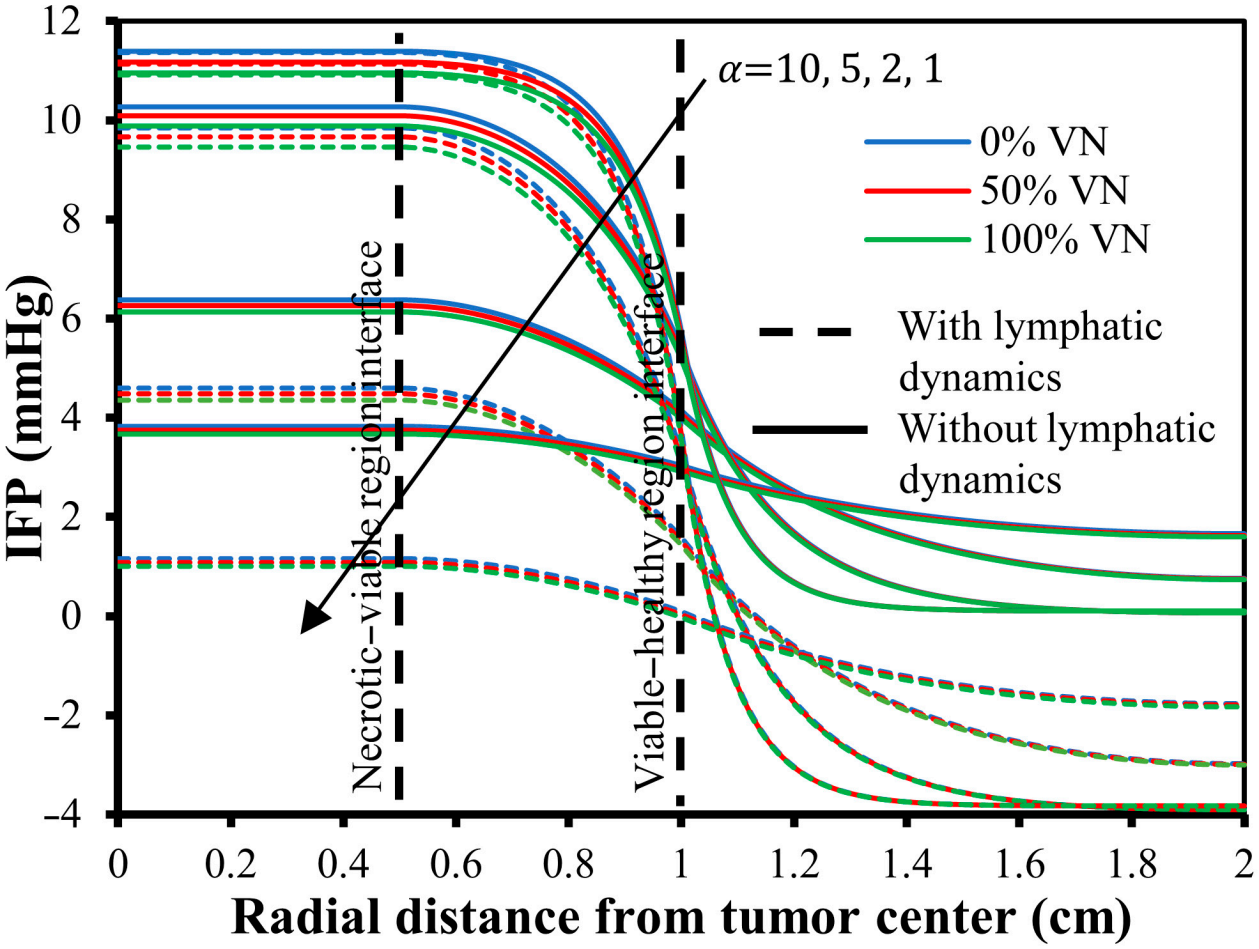
IFP distribution w.r.t. 0%, 50%, and 100% VN with (small dash symbol) and without (solid line) considering functional lymphatics dynamics for different α.

**Fig. 11.**
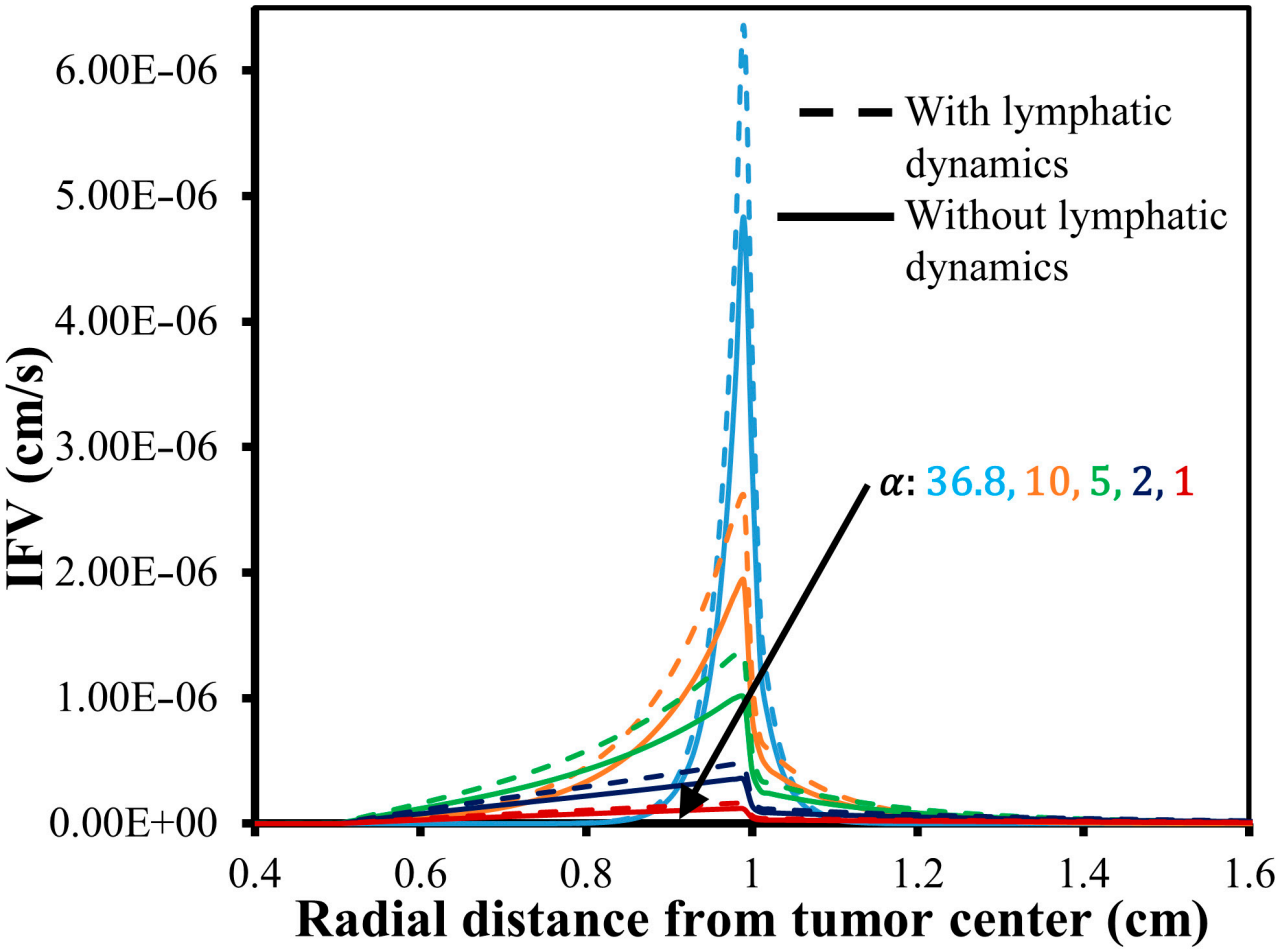
IFV distribution w.r.t. 0% VN with (small dash symbols) and without (solid line) considering functional lymphatics dynamics for different α.

**Fig. 12.**
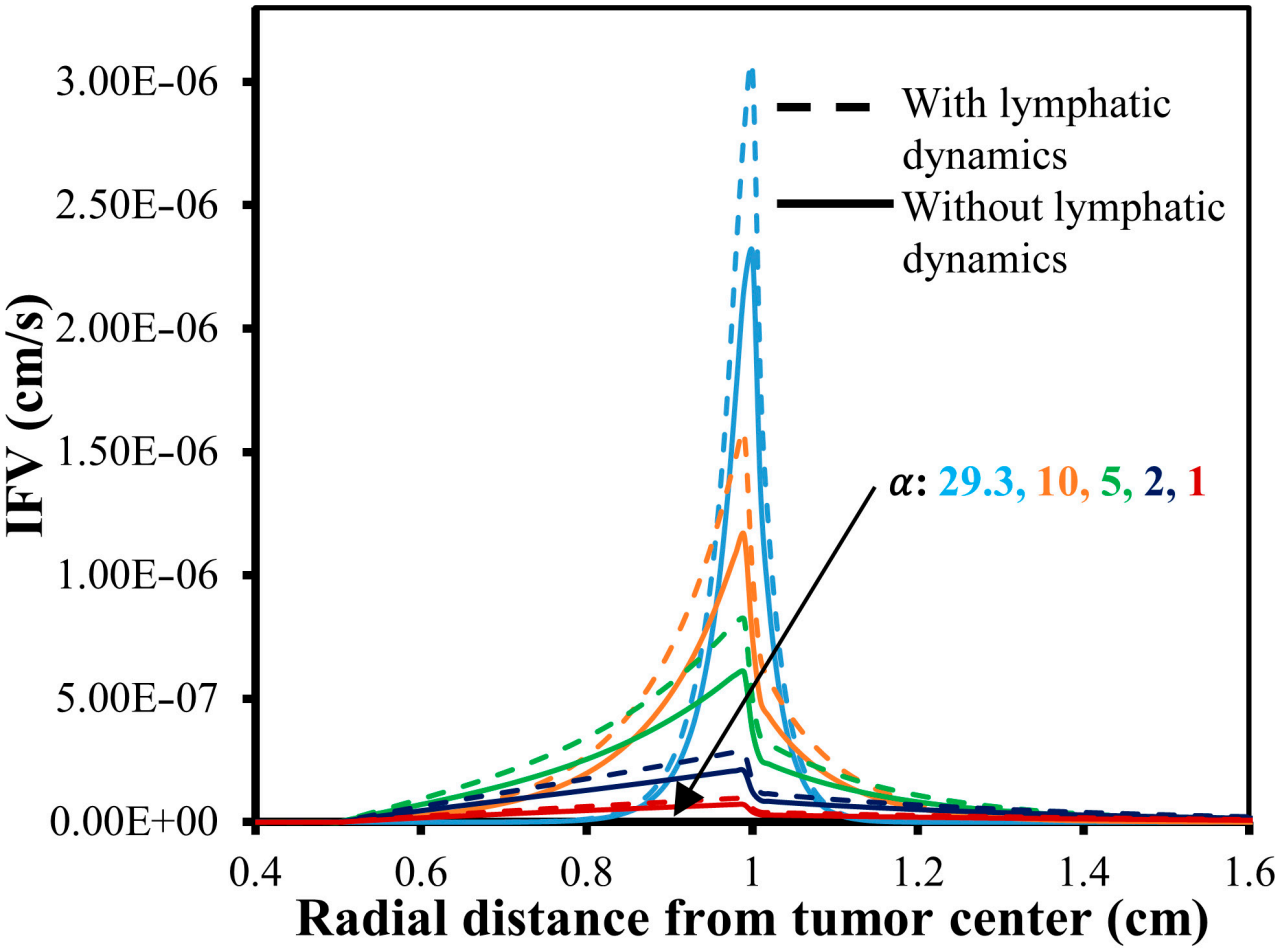
IFV distribution w.r.t. 50% VN with (small dash symbols) and without (solid line) considering functional lymphatic dynamics for different α.

**Fig. 13.**
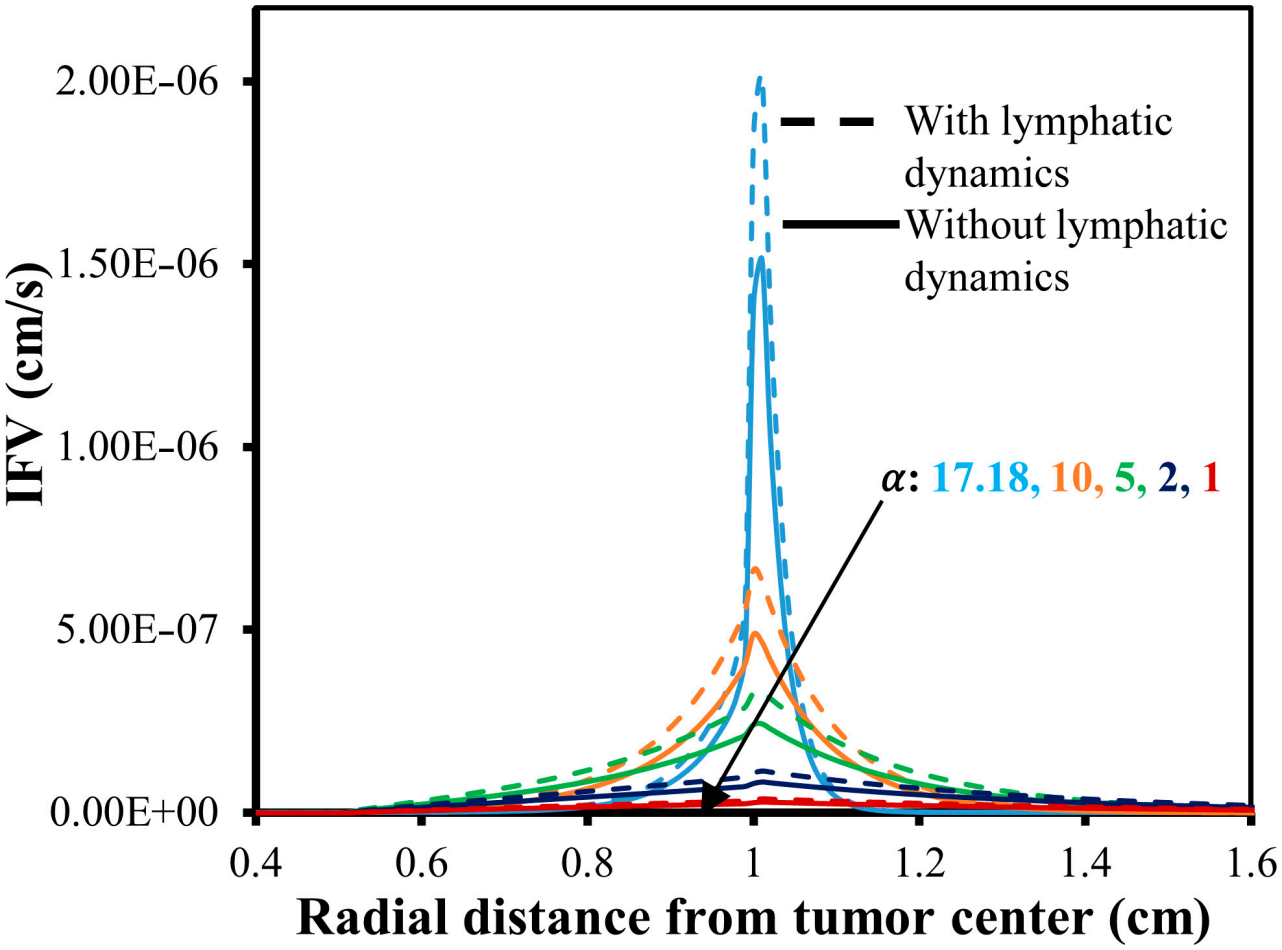
IFV distribution w.r.t. 100% VN with (small dash symbols) and without (solid line) considering functional lymphatics dynamics for different α.

Figures 7 and 8 provide a detailed visualization of the IFP and velocity distribution for various α values, generated using the baseline parameters from Tables 2 and 4 organized into a 3-column layout. The first column in both figures depicts a 2D view of the IFP and IFV distribution relative to 0%VN. These plots reveal the spatial distribution of IFP and IFV within the defined geometry, highlighting trends and variations in the scalar fields. The second column presents the corresponding contour plots for IFP and IFV distributions, showing contour lines that connect points with equal scalar values. These contour plots effectively illustrate constant-value regions within the domain, enhancing the analysis of gradients and transitions in IFP and IFV across the geometry. The third column showcases the 3D visual plots of the IFP and IFV distributions. These plots represent the scalar field solutions derived from solving the PDE model. In these visualizations, the XY plane corresponds to the spatial coordinates, while the Z axis indicates the magnitude of the solution values at respective nodal points. This 3D representation provides an intuitive understanding of the spatial variations and the overall behavior of IFP and IFV within the TME. In order to address variations in IFP and IFV for different degrees of VN (0%, 50%, and 100%) and the presence and absence of lymphatic dynamics, we have provided unidirectional graphs (Figs. 9 to 13) along the transverse line instead of additional 2D and 3D plots. Given the symmetrical geometry and homogeneous nature of each tumor region, the 2D and 3D plots would exhibit similar patterns. The unidirectional graphs comprehensively illustrate the IFP and IFV dynamics for all cases, including both the presence and absence of lymphatic dynamics, effectively capturing the key trends and variations.

Figures 9 to 13 showcase the unidirectional IFP and corresponding IFV distribution along the transverse line. Figure 9 presents the IFP distribution profile employing the baseline parameters from Tables 2 and 4. It is observed that the resulting IFP at the center of the necrotic region shows marginal fluctuations: 11.5, 11.275, and 11.0485 mmHg for 0%, 50%, and 100% VN (with corresponding αvalues36.8,29.3,and17.18), respectively. Notably, these changes in IFP profile are minimal. Moreover, considering the influence of lymphatic dynamics, i.e., non-zero lymphatic hydrostatic pressure in the healthy region, the IFP at the center of the necrotic region remained nearly unchanged, i.e., 11.5, 11.275, and 11.048 mmHg. Also, the alterations in IFP are deemed negligible in the tumor region, while there is negative interstitial pressures in healthy tissue, which is primarily attributed to the effects of functional lymphatic dynamic’s non-zero lymphatic hydrostatic pressure. It can also be observed that the IFP peaks and remains at a constant value of 11.5 mmHg, same as the viable tumor region’s steady-state pressure ($P_{\mathrm{SS}\_\text{viable}}$) within the necrotic and most of the viable tumor region, with a sharp decline near the interface between the viable and healthy regions, eventually stabilizing to $P_{\mathrm{SS}\_\text{healthy}}$ in the healthy tissue region.

Figure 10 shows IFP distribution profiles for different tumor fluid flow resistance parameters (α), corresponding to 0%, 50%, and 100% VN, considering both the case and the presence and absence of functional lymphatic dynamics. This examination of the IFP distribution across different α reveals noteworthy trends. First and foremost, there is a pressure decline as the resistance parameter α decreases. For α exceeding 10, the IFP profile displays minimal variance (compared with Fig. 9), except for slight fluctuations near the interface of the viable and healthy regions. In contrast, for α below 10, the IFP demonstrates more striking changes, with a swifter decline and convergence toward a flatter curve. Particularly for α below 5, the IFP exhibits heightened sensitivity to alterations. The impact due to VN is very small, as it has a marginal impact on the fluid flow profile. With 50% and 100% VN, fluid pressure at the center of the necrotic core decreases by 1.95% and 3.93%, respectively, in the absence of functional lymphatic dynamics. When VN is combined with lymphatic dynamics, there is no notable effect on the overall profile, except a negative pressure profile at the healthy region due to pump-like effects of functional lymphatic dynamics’ non-zero hydrostatic lymphatic pressure.

Darcy’s law presented in Eq. 4 states that fluid velocity is directly proportional to the interstitial pressure gradient. Hence, this creates a pronounced outward interstitial fluid flow at the interface of viable and healthy regions, which causes solute convection to push the solute away from the viable region and into the surrounding healthy tissues. This can reduce the therapeutic effect and increase harm to normal tissues. The high IFP in most parts of the tumor, especially in the non-vascular necrotic core, occurs because the fluid in the necrotic core is trapped and continuously accumulates, thereby being forced to maintain the same pressure as the steady-state pressure of the viable region ($P_{\mathrm{SS}\_\text{viable}}$). This happens because the completely vascularized region is near the tumor periphery, thereby creating considerable back pressure at the tumor core due to the lack of lymphatic vessels to relieve the pressure.

Figures 11 to 13 depict the distribution of the corresponding IFV for 3 distinct scenarios of VN, considering both the presence and absence of lymphatic dynamics across various α. Despite variations in IFV, the overall nature of the 3 graphs remains largely similar. However, as α decreases, there is a noticeable decline in IFV at the interface between the viable and healthy regions, eventually converging toward steeper to nearly flat curves indicative of zero velocity across all regions. Furthermore, it is noted that the IFV exhibits a slightly sharper and higher profile in cases where complete functional lymphatic dynamics are present. This phenomenon can be attributed to the pump-like nature of lymphatic vessels.

In summary, lower α values signify diminished resistance within the interstitium, resulting in a relatively uniform pressure profile. Conversely, elevated α values engender heightened resistance to interstitial fluid transport compared to microvessel flow, culminating in a sharp pressure gradient near the periphery of the viable region. Thus, in simple words, α identifies different patterns at the interface of the viable tumor and the healthy region: larger α results in sharper curves that reflect increased resistance to fluid inflow, whereas smaller α produces flatter curves that signal decreased resistance to fluid flow. Moreover, as IFP converges toward effective pressure ($P_{\mathrm{EF}}$) at the necrotic core, the filtration of fluids and macromolecules from blood vessels diminishes rapidly. However, at the viable region’s outer boundary, where IFP sharply declines and approaches normal tissue pressure, extravasation reaches its peak. Table 5 presents a comparative analysis of the maximum IFP at the center of the necrotic region and the IFV at the interface of viable and healthy regions, across varying degrees of VN (0%, 50%, and 100%) for different α.

**Table 5.** Maximum IFP and IFV attains at 0%, 50%, and 100% VN for different α

α	Without lymphatics dynamics $\left(P_{L}=0\right)$						With lymphatics dynamics $\left(P_{L}=-4\right)$					
	0% VN		50% VN		100% VN		0% VN		50% VN		100% VN	
	IFP	IFV	IFP	IFV	IFP	IFV	IFP	IFV	IFP	IFV	IFP	IFV
36.8, 29.3, 17.18	11.5	0.0484	11.275	0.0231	11.0485	0.0152	11.5	0.0635	11.275	0.0305	11.048	0.0202
15	11.4917	0.0273	11.2669	0.0164	11.0421	0.00728	11.4889	0.0366	11.2641	0.0221	11.0392	0.00987
10	11.3992	0.0194	11.1763	0.0116	10.9531	0.00488	11.3644	0.0261	11.1423	0.0158	10.9182	0.00664
5	10.2765	0.01	10.0893	0.00607	9.8859	0.00243	9.8517	0.0135	9.6694	0.00822	9.4652	0.00330
2	6.3855	0.00352	6.261	0.00208	6.1346	0.000843	4.6071	0.00474	4.4827	0.00282	4.3557	0.00115
1	3.8265	0.00118	3.7535	0.000703	3.6726	0.000282	1.1577	0.00159	1.0853	0.000953	1.0023	0.000385

**Fig. 14.**
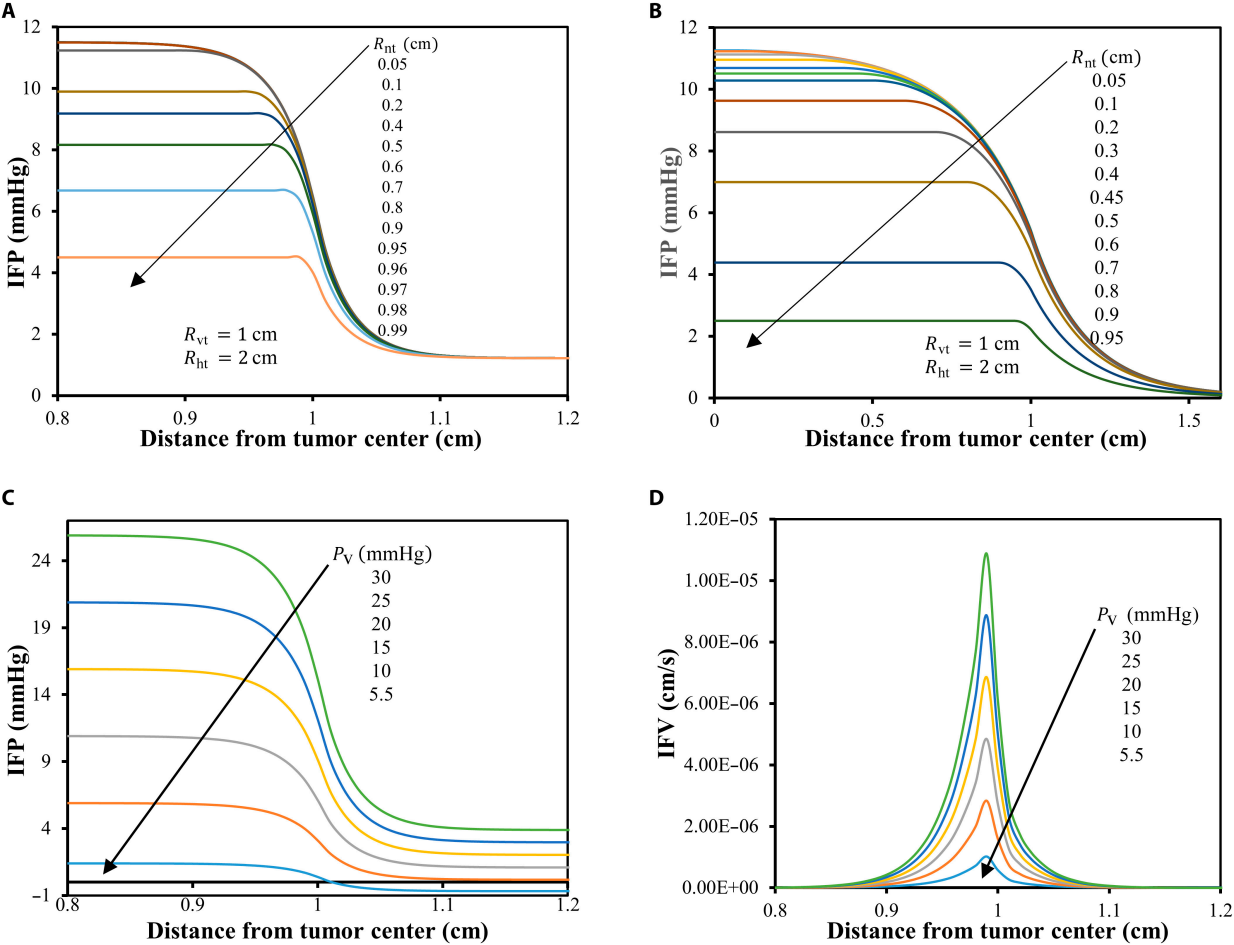
(A and B) IFP distribution for different necrotic size with constant size of viable and healthy region w.r.t. α=36.8 and α=5, respectively. (C and D) IFP and IFV distribution over the parameter $P_{V}$ (microvascular pressure), respectively.

Elevated interstitial pressure is a key factor contributing to the uneven distribution of nanodrug within solid tumors, primarily due to the assumption that nanodrugs move along with the interstitial fluid. In addition to irregular blood supply, high interstitial pressure substantially influences nanodrug transport. This study, therefore, examines the effects of 2 critical physical parameters, varying necrotic sizes, and microvascular pressure on IFP distribution to highlight the impact of the tumor’s heterogeneous nature. Figure 14A and B illustrates the IFP distribution for varying necrotic sizes with a constant viable tumor and healthy region size for α=36.8 and α=5. The findings reveal that an increase in the necrotic radius results in a decrease in the maximum pressure inside the tumor. Interestingly, when the entire tumor is necrotic and devoid of vasculature, the IFP reaches close to the maximum pressure in the healthy region. Conversely, for necrotic radii below a certain threshold, the IFP attains its maximum value, closely aligning with the tumor’s effective pressure, $P_{\mathrm{EF}\_\text{viable}}$. This critical necrotic radius marks a limit beyond which the IFP dynamics undergo considerable changes. Furthermore, it is observed that for higher α, i.e., 36.8, there is a sharp and rapid decline in IFP at the interface between the viable and healthy regions, with nearly the same converged IFP value in the healthy region for each different necrotic radii considered. In contrast, in cases with lowerα, i.e., 5, the IFP profile exhibits a more gradual transition near the outer edge of the viable region, manifesting as a flatter curve. This provides valuable insights into the critical necrotic radius,$\;R_{\mathrm{CN}}$, marking the point from which the maximum pressure at the center of the necrotic core begins to decline. For example, for higher α, i.e., 36.8, the critical necrotic radius $R_{\mathrm{CN}}$ is approximately 0.9 cm and above this, say an increase of necrotic core radius ($R_{\mathrm{nt}}$) from 0.9 to 1 cm, the maximum IFP decreases and tends to approach the pressure observed in the healthy region. Below critical radius, the maximum IFP attains the peak level the same as $\;P_{\mathrm{EF}\_\text{viable}}$. Conversely, for lower α, $R_{\mathrm{CN}}$ exhibits a lower range, typically between 0.5 and 0.6 cm. Furthermore, as the necrotic radius nears that of the viable tumor, the maximum IFP decreases more rapidly. Additionally, in the lower α case, the tumors are entirely devoid of vasculature $\left(R_{\mathrm{nt}}\approx R_{\mathrm{vt}}\right)$, resulting in complete necrosis, with the IFP tending to zero. These findings underscore that delivering nanodrug is more effective to smaller viable regions compared to larger ones that surpass the critical necrotic radius. Figure 14C and D illustrate the IFP and IFV for the different microvascular pressure $\;P_{V}$. There is a consistent decrease in IFP observed across the range of $P_{V}$ values from 30 to 5.5 mmHg. Additionally, it is observed that for $P_{V}=5.5$ mmHg, IFP displays negative values in the healthy region, attributable to the adoption of the boundary condition for pressure in the healthy region. Likewise, for IFV, there is a uniform reduction in maximum velocity at the interface between the viable and healthy regions.

The findings presented above underscore an important discrepancy in IFP distribution between tumor and healthy tissue, with tumor tissue exhibiting substantially higher IFP values while healthy tissue displays markedly lower IFP levels. This disparity can be attributed to distinct tumor characteristics, such as the absence of vasculature in the necrotic region and dysfunctional lymphatic drainage systems in the viable region. At the interface between viable and healthy regions, a pressure gradient prompts the exchange of interstitial fluid within a confined area, resulting in an observable increase in velocity. As this traverses from the tumor’s core toward its periphery, a marked decline in IFP is observed. This reduction in IFP is primarily attributed to the migration of fluid from the interstitium into the bloodstream, facilitated by the pressure gradient existing between microvessel pressure and IFP. Similarly, results reveal analogous patterns in the distribution of IFV in the transverse direction. The simulated maximum pressure in this study is 11.5 mmHg (~1,533Pa) at the center of the necrotic core for the 0% VN case, showing strong agreement with the benchmark FES study by Mahesh et al. [15] and the experimental findings of Baxter and Jain [7], which reported a similar IFP of approximately 1,533 Pa. Additionally, the estimated maximum and minimum IFP values in this study for tumor and normal tissues are 11.5 mmHg (~1,533Pa) and 1.211 mmHg (~162Pa), respectively, aligning with experimental literature [61,62], which reported IFP values ranging from 586 Pa (~4.4mmHg) to 4,200 Pa (~31.50 mmHg) for tumor tissue and −400 Pa (~−3 mmHg) to 800 Pa (~6 mmHg) for normal tissue. Furthermore, the study’s IFP and IFV results are consistent with both experimental and computational findings [6,7,13,15,63]. This elevated IFP combined with irregular blood flow greatly affects how drugs are distributed within solid tumors. One approach to reduce IFP involves altering osmotic or vascular pressures. Lowering the surface area of tumor blood vessels could decrease fluid filtration, but it may also reduce solute filtration. Additionally, increasing tissue hydraulic conductivity K could reduce pressure generated for a certain amount of fluid loss. Various methods, such as radiation or immunotoxins to kill cancer cells or using a protease like hyaluronidase to disrupt the interstitial matrix, could raise K [64].

### Nanodrug distribution in the interstitial space

In the preceding section, an investigation is conducted into the IFP and IFV within the tumor with an aim to elucidate the role of convection mechanisms in the distribution of nano-sized solutes. The distribution patterns of nanodrugs across various regions also need to be examined to evaluate their impact on treatment response. Importantly, the investigation is focused on the distribution mechanism of different NPs as nano therapeutic drug carriers used for cancer treatment and compared them with the well-known conventional drug, doxorubicin, thereby enriching the novelty of the current research. The model is also used to evaluate the impact of important physical parameters, such as solute degradation rates and diffusion coefficient, which have a striking impact on how therapeutic agents are distributed. Given the superior therapeutic outcomes associated with intratumoral injections compared to other approaches, it is pertinent to highlight the focus of the study on the post-injection distribution of nanodrugs within tumors. Here, the study assumes an initial administration of NP dose (carrying therapeutic drug) dissolved in water and injected uniformly into the necrotic region. The initial dose preparation from previous studies [15,65,66] is considered, where the particle dose of 4 mg/cc of tumor is dissolved in 0.1 ml of water for injection.

**Fig. 15.**
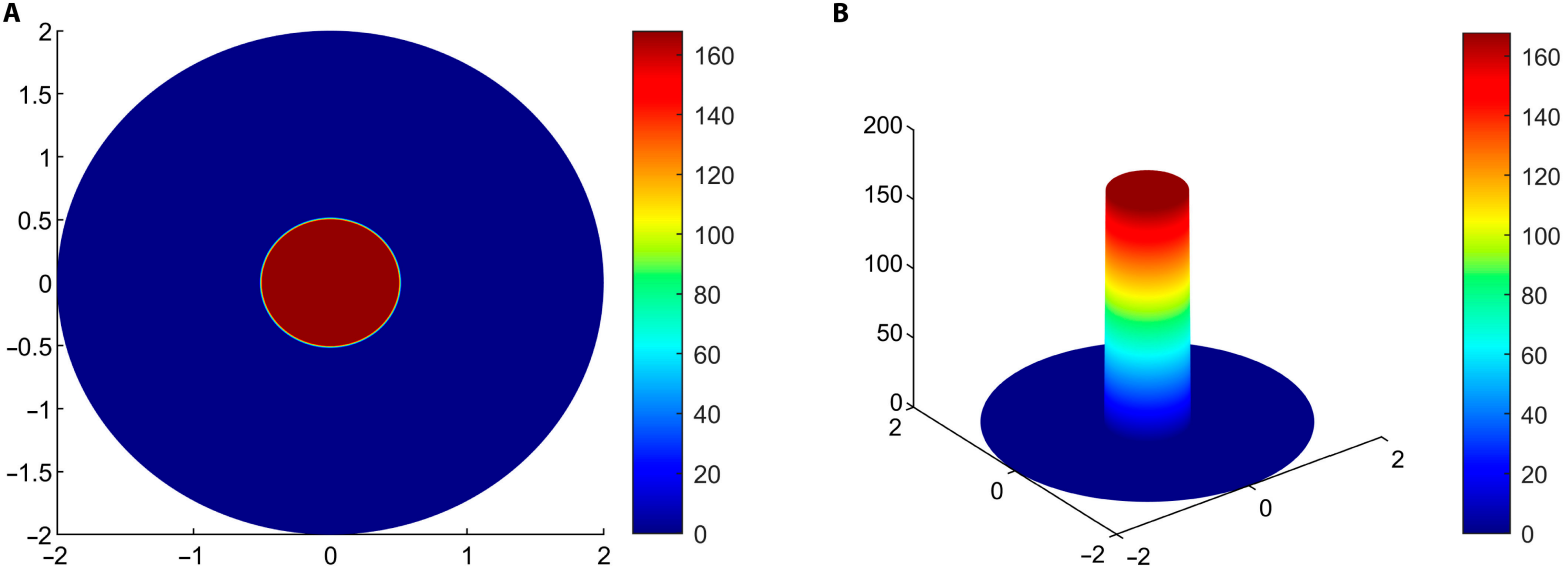
(A) 2D representation of initial concentration $\left(\mathrm{mg}/\mathrm{cc}\right)$ after intratumoral injection in the necrotic region. (B) Initial concentration distribution is visualized as a 3D plot height, reconstructing the axisymmetric solution into a cylindrical shape.

The spatial distribution of nanodrug within tumor tissues is determined by simulating the transient CDR equation. In this study, simulations are conducted to ascertain the distribution of NPs with sizes of 10, 20, 30, 50, and 100 nm. Figure 15A and B offer visual representations of the initial solute concentration of nanodrug within the interstitial space after the uniform intratumoral injection in the necrotic region, depicted in 2D and 3D formats, respectively.

**Fig. 16.**
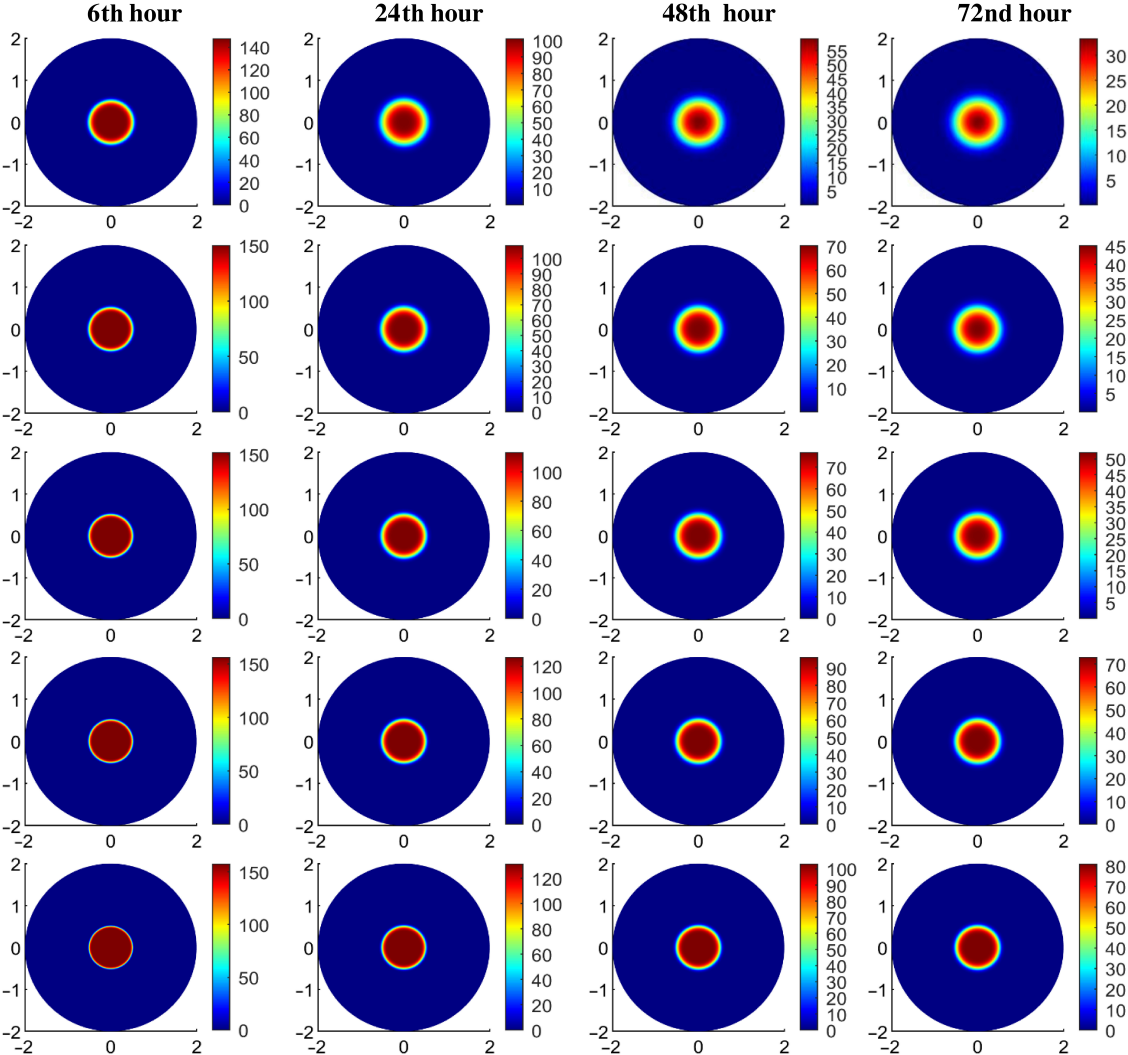
Spatial distribution of particle concentration $\left(\mathrm{mg}/\mathrm{cc}\right)$ within the tumor of 10-, 20-, 30-, 50-, and 100-nm (row wise) sizes at different times following the intratumoral injection, respectively.

**Fig. 17.**
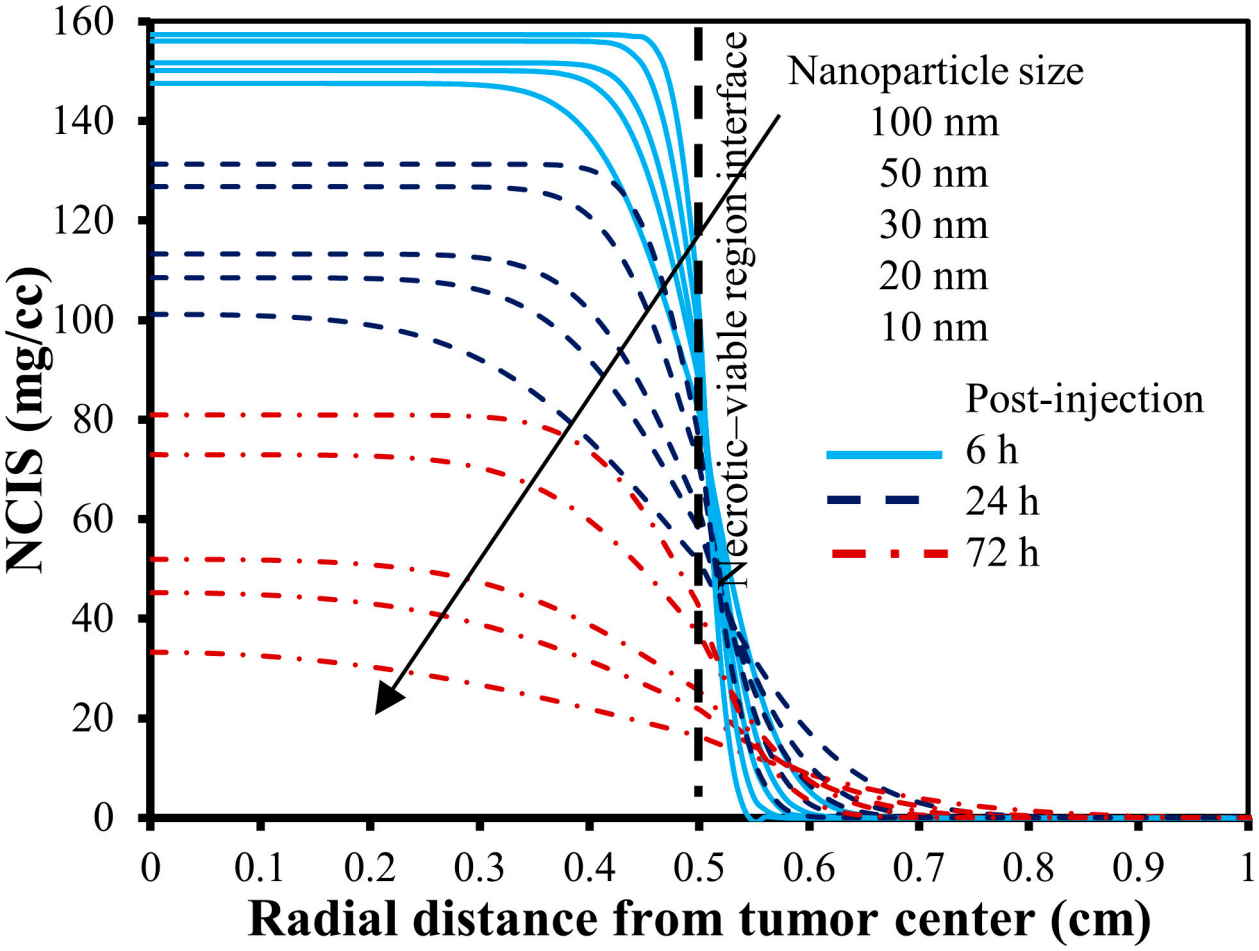
Radial–temporal depiction of particle concentration within the tumor of different sizes at different post-injection times.

Figure 16 illustrates the spatial distribution of NPs of varying sizes at the 6th, 24th, 48th, and 72nd hour post-intratumoral injection. First, let us observe the first row of Fig. 16, with 2D plots showing the distribution of 10-nm NPs in the tissues at different times after intratumoral injection. It is observed that as time passes, the NPs spread out from the injection site, causing the concentration of nanodrug in that area to decrease. This happens because the NPs move into the surrounding tumor region due to a concentration gradient and the movement of interstitial fluid. The same trend is seen for all particle sizes studied. The low IFV signifies that the contribution of convective transport from interstitial fluid flow is minimal, and diffusion remains the primary mechanism for NP transport in tumors. Equation 18 highlights the inverse relationship between diffusion and NP size, indicating that larger NPs exhibit weaker diffusion. Given that NP distribution is primarily governed by diffusion, it is evident from the figure that larger NPs experience slower diffusion from the necrotic region to the viable region, resulting in a gradual decrease in concentration over time for larger NPs. Thereby, due to their slower removal rate from the interstitial space, a higher concentration is maintained within the tissue for prolonged durations. Conversely, smaller sized NPs show quite opposite trends. Moreover, observing the 2D illustration in Fig. 16, it becomes apparent that the distribution of NP concentration exhibits symmetry in all directions. The time given for diffusion after the injection will also change how nanodrugs spread inside tumors. To enhance clarity, Fig. 17 displays the unidirectional distribution of NPs of various sizes over time by specifically focusing on NP distribution along the transverse line. It compares the distribution of NPs ranging from 10 to 100 nm in size at 3 distinct time points following intratumoral injection, i.e., 6, 24, and 72 h. The results clearly illustrate that smaller NPs are degrading more rapidly, whereas larger particles persist for longer durations in the necrotic region with minimal penetration in the viable region. It can also be observed that NPs sized between 10 and 30 nm exhibit a similar concentration distribution trend, while those sized between 50 and 100 nm show a closer pattern, indicating a critical transition in distribution pattern for an NP size between 30 and 50 nm. In order to achieve optimal efficacy, it is crucial for NPs to penetrate the viable region before being washed out through transvascular exchange [6]. This requires maintaining a sufficiently high concentration of nanodrug within the tumor tissue for an extended period. Physically, this involves ensuring that NPs are small enough to diffuse effectively through the extracellular matrix but large enough to avoid rapid degradation from the tumor via the vasculature. Additionally, the balance between diffusion and convection forces plays a vital role; effective delivery must overcome the outward interstitial fluid flow that can push nanodrug toward normal tissues. By optimizing these factors, the therapeutic agents carried by the NPs can remain within the tumor long enough to exert their intended effects. Thus, the study concludes that smaller particles diffuse more easily into the viable tumor region but are quickly degraded due to nano-bio interactions, resulting in lower concentrations there. Larger particles tend to remain in the necrotic core even long after injection. For instance, 10-nm particles diffuse the fastest but are swiftly degraded, leading to insufficient therapeutic levels in the viable tumor region. Particles larger than 30 nm do not show notable concentrations in the viable region due to their lower diffusion rates. This underscores the importance of NP size, suggesting an optimal range between 15 and 30 nm for enhanced treatment efficacy, optimal drug transport, and hyperthermia applications. The NP concentration distribution results depicted in these figures are consistent with findings from previous studies [7,15,60,67].

### Comparison of nanocarriers to conventional doxorubicin

**Fig. 18.**
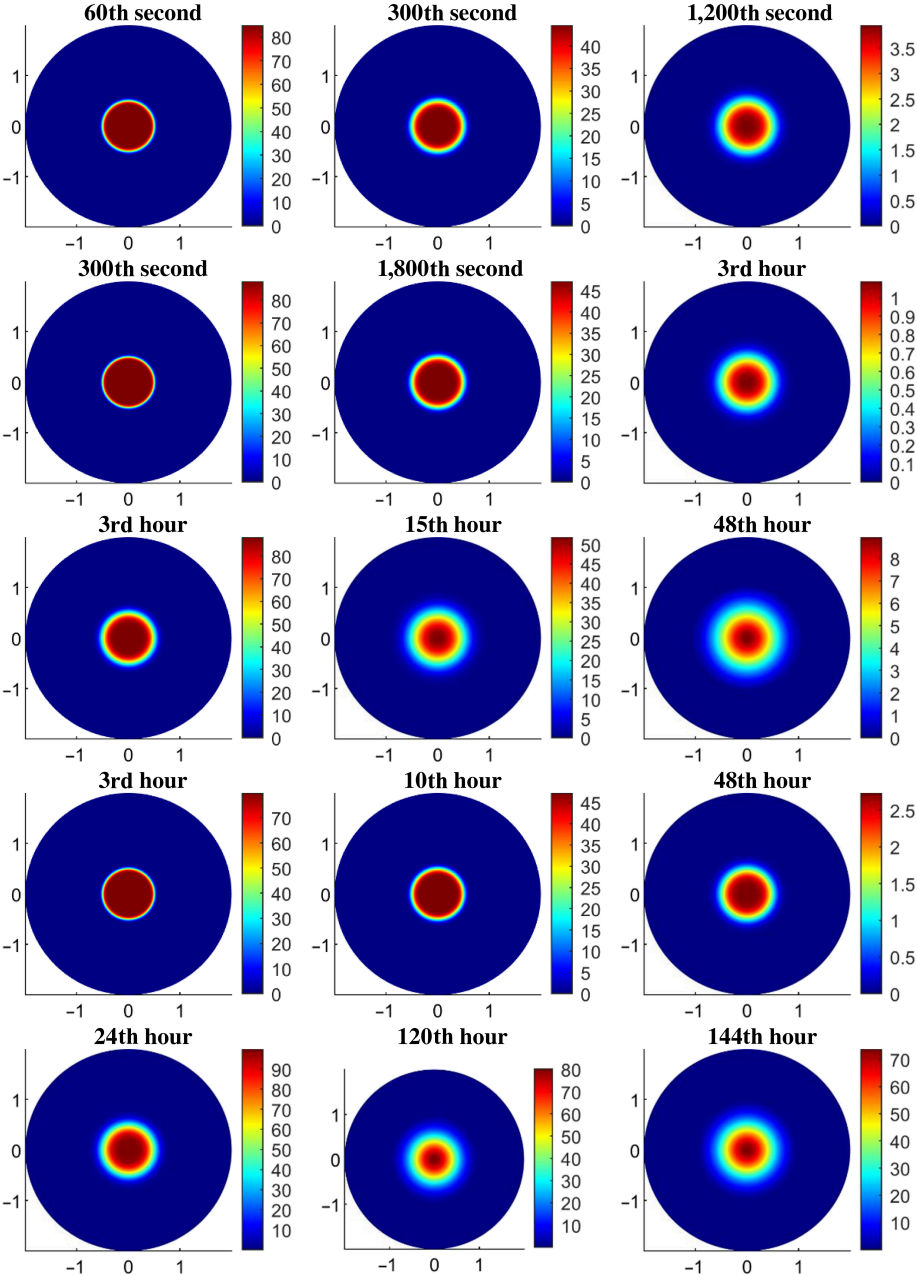
2D portrayal of distribution of conventional doxorubicin, dextran, liposomal, PEG-coated gold, and magnetic (row wise) at different times after the intratumoral injection (in mg/cc).

**Fig. 19.**
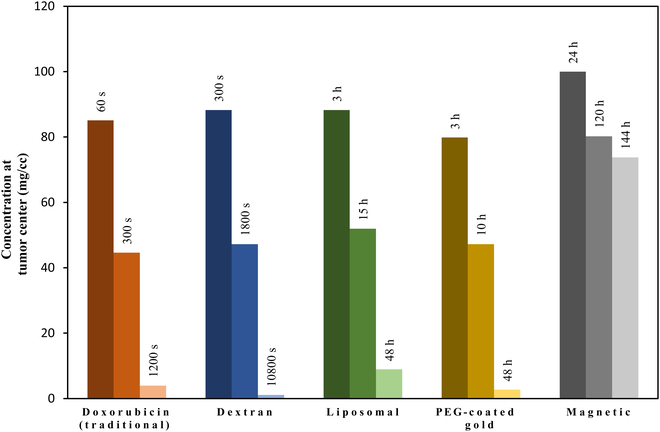
Bar chart representating the comparison for the temporal evolution of nanodrug concentration (mg/cc) at the tumor center followed by the intratumoral injection.

The use of NPs as carriers for antitumor drugs is generally preferable to molecular chemotherapeutic agents due to their higher efficacy against tumor cells and reduced harm to normal tissues. The model is employed to analyze and compare the distribution of various nanodrug carriers against the conventional chemotherapeutic agent doxorubicin, quantifying therapeutic effects. Selecting nanodrug carriers requires biocompatibility, high drug loading, targeted delivery, stability, biodegradability, optimal size, surface properties, controlled release, and responsiveness to stimuli for effective cancer treatment [68]. Table 6 categorizes these nanodrugs based on the aforementioned characteristics and their clinical status, while Table 7 provides parameter values for both tumor and healthy tissues, sourced from existing literature. In order to facilitate comparison of the concentration distribution of these nanodrug carriers, the study has assumed an initial uniform NP concentration of 100 mg/cc at the necrotic region. Figure 18 displays the 2D spatial distribution of doxorubicin and other nanodrug carriers (dextran, liposomal, PEG-coated gold, and magnetic) considered in this study. It is evident that the conventional drug doxorubicin is washed out within 30 min after intratumoral injection, mainly due to its high interstitial diffusivity and degradation rate. In contrast, magnetic nanodrugs exhibit a considerably longer retention time at the injection site, attributable to their low diffusivity and degradation rate. The bar chart in Fig. 19 illustrates a comparison of the temporal evolution of all these nanodrug concentrations at the tumor center. It can be observed that doxorubicin and dextran display similar behavior, being cleared from the interstitial space within 1 to 3 h. Liposomal and PEG-coated gold NPs exhibit almost a similar behavior, while magnetic nanodrugs demonstrate a completely different pattern. Hence, the findings suggest that assessing total solute exposure over time relies substantially on 2 key factors: effective diffusivity power and degradation rate constant. These factors determine how long a solute concentration persists in the tumor region, its penetration from the necrotic to the viable region, and its degradation due to nano-bio complex reactions. This reduced retention and solute exposure suggest inadequate delivery of anticancer agents to the viable tumor region, potentially leaving cancer cells untreated. Hence, alternative treatment methods such as surgery or thermal therapy may be necessary to enhance cancer treatment effectiveness. In summary, this study suggests that larger NPs, such as liposomal, gold-coated ones, or magnetic may offer more effective therapeutic outcomes in the tumor region compared to directly injecting molecular anticancer agents like doxorubicin.

**Table 6.** Physiological characteristics of doxorubicin and nanocarriers

Nanoparticle	Advantages	Disadvantages	Status	Ref.
Doxorubicin (traditional drug)	• Tumor stimuli responsive drug release capacity • Water soluble and photosensitive • High drug loading efficiency • Combinational therapy ability	• Off-target side effects • Severe inflammatory responses • Hydrophobicity • Dysfunction the immune cells • Faster degradation	Clinically approved in 1974	[78]
Dextran	• Slow release and water soluble • Large drug loading capacity • Biocompatible and biodegradable • Convenient absorption • Achieve long circulation in blood • Programmed to respond to natural stimuli • Engineered for tumor imaging	• Shows side effects like liver damage, thrombocytopenia • Rapid clearance • Manufacturing challenge due to lack of understanding of the compositions and interacted mechanism with tissue	Preclinical stage	[79]
Liposomal	• Biodegradable and biocompatible • Hydrophilic and lipophilic in nature • Enhancement in half-life of drug • Low immunogenicity • Reduce off target toxicity • Stimuli responsive • Improved tissue stability and drug stability	• Low entrapment • High macrophage and reticuloendothelial uptake rate • Accelerated blood clearance • Lack of specific targeting and deposition • Show potential toxicity	Clinically approved in 1995	[80]
PEG-coated gold	• Reduced protein absorption • Longer circulation time in bloodstream • Improved delivery and stability of drugs • Presence of tunable optical properties • Biocompatible • External stimuli and internal stimuli responsive	• Unclear toxicity in humans • Immunogenicity • Incomplete clearance; lack of assessment of the under the pharmacological point of view • Sometime allergic and potentially toxic in nature • High cost of production	Under clinical trials	[81]
Magnetic	• Contrast agents in magnetic resonance imaging • Molecular imaging • Offer high magnetic moments and surface area-to-volume ratio • Can improve the sensitivity of biosensors and diagnostic tools • Magnetic hyperthermia; targeted drug delivery • Longer retention stability	• Low biocompatible • Insufficient magnetic strength • Low drug loading capacity • Limited penetration • Difficulty in tuning their size for special biomedical applications	Under clinical trials	[82]

**Table 7.** Parameters for doxorubicin and different nanocarriers in tumor and healthy region

Nanoparticle no.	Description	Interstitial diffusivity $\left(D_{\mathrm{EF}},\frac{cm^{2}}{s}\right)$		Degradation rate $\left(\tau_{\deg},\frac{1}{s}\right)$		Ref.
		Tumor	Normal	Tumor	Normal	
D-1	Doxorubicin (traditional chemotherapeutic drug)	$3.4\times 10^{-6}$	$1.58\times 10^{-6}$	$2.69\times 10^{-3}$	$2.689\times 10^{-3}$	[34]
D-2	Dextran (with molecular weight 70 kDa)	$1.4\times 10^{-7}$	$5\times 10^{-9}$	$4.17\times 10^{-4}$	$2.085\times 10^{-4}$	[34]
D-3	Liposomal nanoparticles	$3.35\times 10^{-7}$	$2.4\times 10^{-9}$	$1.16\times 10^{-5}$	$1.157\times 10^{-5}$	[83]
D-4	PEG-coated gold nanoparticle (PEG-AuNPs)	$4.40\times 10^{-8}$	$1.2\times 10^{-8}$	$2.08\times 10^{-5}$	$2.083\times 10^{-5}$	[13,50]
D-5	Magnetic nanoparticle ($Fe_{3}O_{4}$)	$8.89\times 10^{-8}$	$4.17\times 10^{-8}$	$3\times 10^{-16}$	$3\times 10^{-16}$	[19]

### Effect of effective diffusivity and degradation rate

**Fig. 20.**
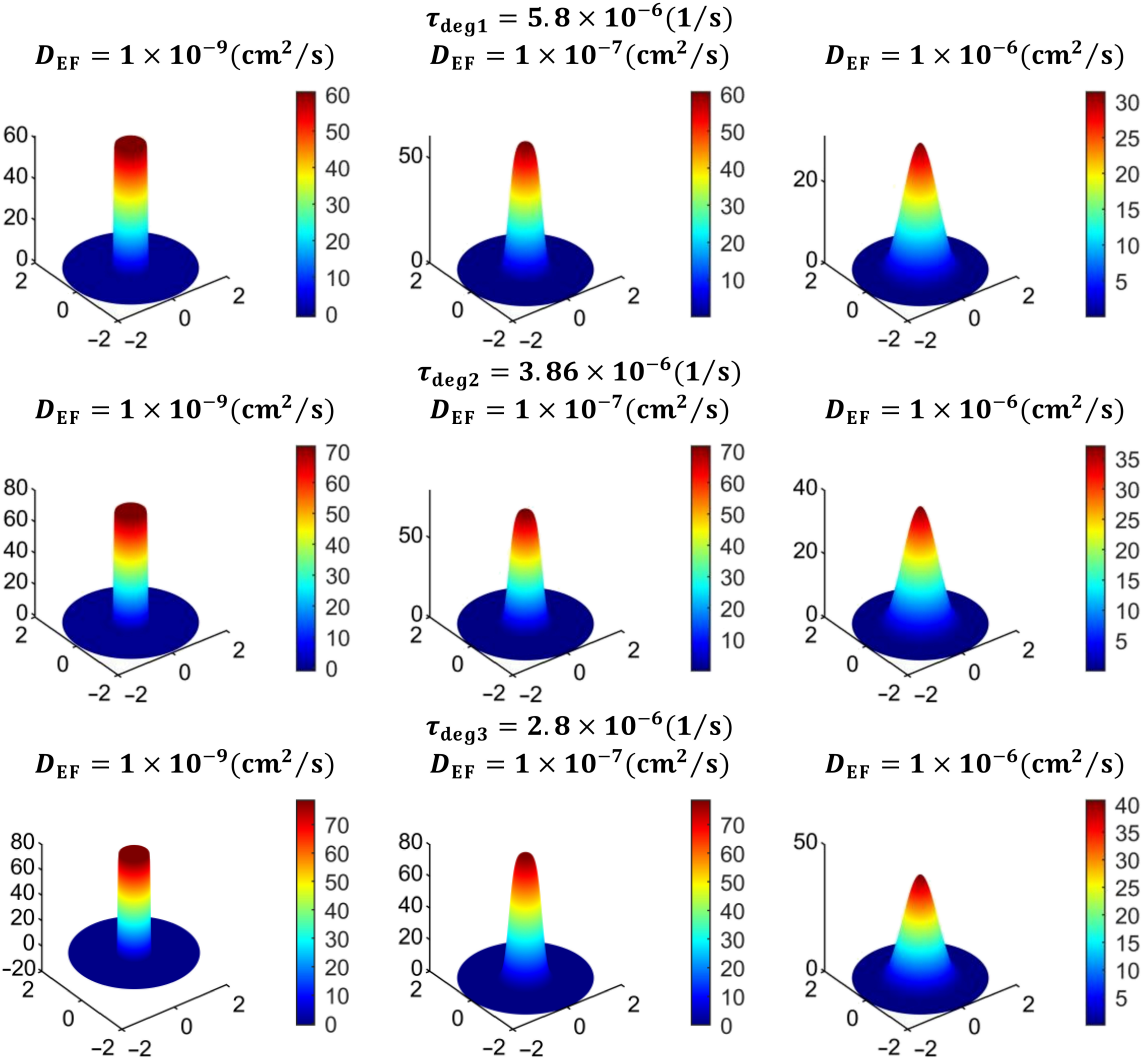
3D plots illustrating the spatial distribution of nanoparticle concentration (in mg/cc) at various diffusivity and degradation rates, 24 h post-intratumoral injection.

**Fig. 21.**
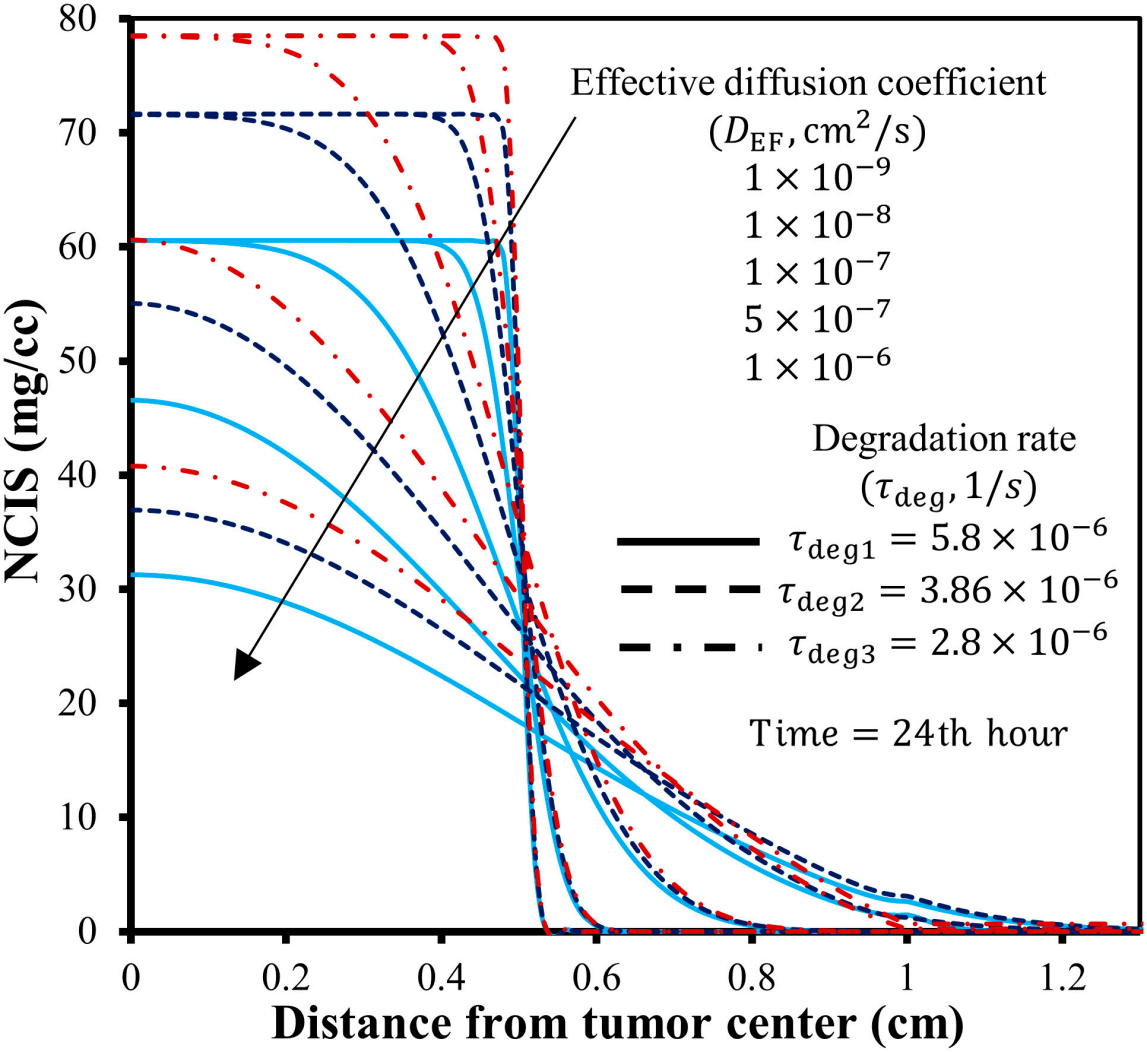
Radial–temporal depiction of nanodrug concentration (in mg/cc) at various diffusivity and degradation rates 24 h post-intratumoral injection.

In this section, the study investigates the effects of various effective diffusivities across 3 distinct degradation rates on NP concentration distribution. Figure 20 illustrates the NP concentration distribution 24 h after intratumoral injection, shown as a 3D plot for varying effective diffusion coefficients and 3 distinct degradation rates. The 3D visualization reconstructs the axisymmetric solution into a cylindrical shape, using solution values (magnitude) to represent plot heights. The figure reveals a correlation between the diffusion coefficient and degradation rate, with higher degradation rates and diffusion coefficient results for faster nanodrug degradation from the tumor. Specifically, the radial–temporal evolution of nanodrug concentration at various diffusivities and degradation rates 24 h post-intratumoral injection is further clarified using the unidirectional representation along the transverse line (Fig. 21). The figure illustrates that with higher degradation rates $\left(\tau_{\deg1}\right)$ and diffusion coefficients, the peak nanodrug concentration at the tumor center decreases by approximately 70% within 24 h post-injection. Conversely, with a decrease in degradation rate $\;\left(\tau_{\deg2}\right)$, nanodrug concentration persists for a longer duration within the tumor, leading to improved penetration into the viable region. However, at the lowest degradation rate $\left(\tau_{\deg3}\right)$ considered in this study, particles remain concentrated at the injected region initially, particularly with lower diffusion coefficients. Although higher diffusion coefficients allow particles to move toward the viable region, the concentration is lower compared to that at $\;\tau_{\deg2}$, in the viable region. This observation underscores the potential enhancement of nanodrug delivery efficacy through particle nano-engineering, with careful consideration of parameters like diffusion coefficient and degradation rate.

### Implications for NP design and clinical translation

In cancer nanomedicine, NPs are engineered to enhance drug delivery to tumors while minimizing systemic toxicity. However, optimizing NP design remains challenging due to the complex interplay of tumor heterogeneity, IFP, and NP properties such as size, surface functionalization, diffusivity, and degradation rate [16]. Computational models provide a powerful framework to predict NP behavior, refine experimental strategies, and optimize therapeutic outcomes by identifying key design parameters.

The model developed in this study addresses these challenges by simulating interstitial fluid flow, solute transport, and NP dynamics within solid tumors, offering valuable guidance for NP design. For instance, our investigation indicates that NPs in the 15- to 30-nm range exhibit optimal therapeutic efficacy and concentration-dependent hyperthermia potential, providing a clear target for NP synthesis. Additionally, the superior performance of liposomal, PEG-coated gold, and magnetic-based nanodrug carriers over conventional agents like doxorubicin highlights their potential for clinical translation, as they offer sustained drug distribution and reduce challenges posed by elevated IFP. By tuning NP properties such as diffusivity and degradation rates, drug delivery precision can be enhanced, reducing off-target effects. Furthermore, our model’s ability to predict the influence of TME parameters such as fluid flow resistance (α) and necrotic radius ($R_{\mathrm{nt}}$) on NP transport provides a basis for patient-specific nanomedicine design, tailored to individual tumor characteristics. For instance, the study found that delivering nanodrugs is more effective to smaller viable tumor regions compared to larger ones that exceed the critical necrotic radius ($R_{\mathrm{CN}}$). These insights not only improve our understanding of NP behavior in tumors but also facilitate the development of next-generation nanomedicines with improved clinical applicability. While this study focuses on mathematical and computational predictions, future work aims to collaborate with experimentalists and industrial partners to validate these results and accelerate their translation into clinical practice.

### Limitations and future improvements

While the proposed model provides valuable insights into interstitial fluid flow and nanodrug transport in solid tumors, it has certain limitations that need to be addressed in the future. First, the model assumes a static and homogeneously distributed vasculature, treating the tumor microvascular and lymphatic network as distributed source and sink terms rather than explicitly modeling the capillary network [69,70]. This simplification overlooks the influence of geometric features of tumor vasculature on drug delivery, such as vessel density and branching patterns. Additionally, the model considers vascular density (S/V) as constant for each tumor region, which may not accurately reflect the dynamic and heterogeneous nature of real tumors [13]. Future work could incorporate dynamic vasculature, including angiogenesis and vascular collapse, to better capture the evolving TME. Second, the diffusion coefficient model accounts for particle size and tissue porosity but does not include the effects of NP shape, surface characteristics, or NP-extracellular space molecule interactions [71]. Incorporating these factors could provide a more comprehensive understanding of NP diffusion and stability. Third, the model considers immune system interactions or cellular uptake mechanisms as a constant rate of degradation, which is not realistic in nature, and may vary with time [72]. Fourth, the current framework relies on idealized tumor geometries and homogeneous material properties, which may not fully capture the heterogeneity observed in real tumors. Future work could integrate patient-specific imaging data, such as MRI or CT scans, to create more realistic and personalized models. Addressing these factors could further enhance the model’s predictive capabilities. Finally, while the use of FES ensures computational efficiency, further optimization could be achieved by leveraging parallel computing or machine learning techniques to handle larger and more complex datasets [73]. These improvements would enhance the model’s accuracy and scalability, facilitating its translation into clinical applications, such as personalized nanomedicine and treatment planning.

## Conclusion

Motivated by advancements in nanodrug delivery technologies for cancer treatment, this study deepens the understanding through computational FE simulations of fluid flow and nanodrug transport in solid tumors. FEM has been implemented to simulate and analyze the mathematical framework of the model. The study observes that numerical results predicted here are consistent with those from previous experimental and computational studies [7,15,60,67]. The major findings of the investigation can be summarized as follows:

• IFP peaks at the necrotic core and extends throughout the viable region, declines sharply at the viable–healthy interface, and stabilizes in healthy tissue, while IFV remains nearly zero and uniform except for a surge at the viable–healthy interface.
• A decrease in the fluid flow resistance parameter (α) reduces IFP and leads to a decline in IFV at tumor boundary, which eventually flattens and converges to zero.
• VN minimally affects fluid flow, causing slight pressure reductions in necrotic and viable regions, while complete lymphatic dynamics have a negligible impact except for a negative pressure profile in the healthy region.
• Varying the necrotic radius alters the maximum tumor IFP: below the critical radius $\;R_{\mathrm{CN}}$, IFP peaks and stabilizes, while expansion reduces it.
• With higher α, the critical necrotic radius $R_{\mathrm{CN}}$ increases, while lower α values reduce $\;R_{\mathrm{CN}}$. When the necrotic radius is below the critical threshold, the maximum IFP peaks and aligns with the tumor’s effective pressure $\;P_{\mathrm{EF}}$.
• As the necrotic radius nears that of the viable tumor, i.e., tumors without vasculature, the maximum IFP decreases rapidly, dropping to levels found in healthy tissue.
• Nanodrug delivery is more effective in smaller viable regions than in larger ones exceeding the critical necrotic radius.
• Smaller sized NPs $\left(\sim 10\;\mathrm{nm}\right)$ diffuse faster but face degradation limitations by vasculature extravasation. Conversely, larger NPs $\left(>30\;\mathrm{nm}\right)$ tend to stay at the injection site, lasting in tumors longer.
• Liposomal, PEG-coated gold, and magnetic variants provide therapeutic effects for over 48 h, while doxorubicin is eliminated in less than an hour, potentially yielding better tumor-targeted outcomes.
• NP delivery efficacy can be enhanced through precise particle nano-engineering, involving careful consideration of factors like diffusion coefficient and degradation rate.

In the future, the computational framework and findings of this study can be used to extend the model’s clinical relevance, translational potential, and nanodrug carrier design, as discussed in the “Implications for NP design and clinical translation” section. For instance, integrating concentration-dependent bio-heat transfer dynamics [38] would enable the evaluation of hyperthermia treatments, especially with thermo-responsive NPs, which play a crucial role in controlled drug release and targeted thermal therapy. Incorporating patient-specific imaging data can facilitate personalized treatment simulations, optimizing dosage, treatment frequency, and injection strategies. Additionally, further development to include drug release kinetics [19] and multi-site injection approaches [38] could enhance therapeutic coverage across the tumor. Moreover, the model can be refined further by incorporating all the improvements discussed in the “Limitations and future improvements” section, such as dynamic vasculature, more detailed NP interactions, and accurate diffusion coefficient. Additionally, extending the model to explore combination therapies such as radiation or immunotherapy could provide deeper insights into optimizing multimodal cancer treatments [74]. Finally, collaborative experimental validation both in vitro and in vivo will be critical to refine model parameters and accelerate the translation of these computational insights into effective, patient-specific nanomedicine therapies.

## Data Availability

Data will be made available on request.
